# A green, economical synthesis of β-ketonitriles and trifunctionalized building blocks from esters and lactones

**DOI:** 10.3762/bjoc.15.287

**Published:** 2019-12-06

**Authors:** Daniel P Pienaar, Kamogelo R Butsi, Amanda L Rousseau, Dean Brady

**Affiliations:** 1 Molecular Sciences Institute, School of Chemistry, University of the Witwatersrand, PO Wits 2050, Johannesburg, South Africa

**Keywords:** acylation, β-ketonitrile, enolizable, trifunctionalized, sustainable

## Abstract

The acylation of the acetonitrile anion with lactones and esters in ethereal solvents was successfully exploited using inexpensive KO*t*-Bu to obtain a variety of β-ketonitriles and trifunctionalized building blocks, including useful *O*-unprotected diols. It was discovered that lactones react to produce the corresponding derivatized cyclic hemiketals. Furthermore, the addition of a catalytic amount of isopropanol, or 18-crown-6, was necessary to facilitate the reaction and to reduce side-product formation under ambient conditions.

## Introduction

β-Ketonitriles represent highly versatile intermediates for the synthesis of heteroaromatic compounds and a wide variety of pharmaceuticals. A recent review by Kiyokawa et al. summarizes the applications of these valuable compounds and the abundant methods that have been developed over recent decades to prepare them [1], most of which still involve environmentally unfriendly transition-metal-based reactions. Acylations of in situ-generated nitrile anions with esters to produce β-ketonitriles were first reported long ago, for example by using sodium methoxide [2], sodium ethoxide [3] or sodium amide [4–5]. The reaction was found to proceed more efficiently when using sodium amide as the base [5], although the inherent risks of employing explosive sodium amide in synthesis are well known [6] and amidine side-product formation was observed at times through reaction of the nucleophilic amide base with nitrile. Furthermore, clearly two equivalents of base (and nitrile) were necessary to drive the reactions to completion as the acylated product is more acidic than the nitrile starting material. More recently, ester or Weinreb amide reactions with acetonitrile using lithium bases at low temperature [7], or similarly NaH at high temperatures [8], have been reported as feasible alternatives with varied success and usually a lack of general applicability (e.g., enolizable esters and ketone products may react undesirably under these highly basic reaction conditions). Furthermore, these methods constitute either expensive, less scalable procedures employing an excess of reagents, hazardous, flammable, or toxic reagents and high temperatures are required, which, in turn, may be detrimental to more highly functionalized starting materials. Nevertheless, this reaction under milder conditions is a valuable C–C bond-forming reaction for the preparation of β-substituted carboxylic acid derivatives, in general, from cheap commercially available esters, and as such merited further investigation.

**Scheme 1:**
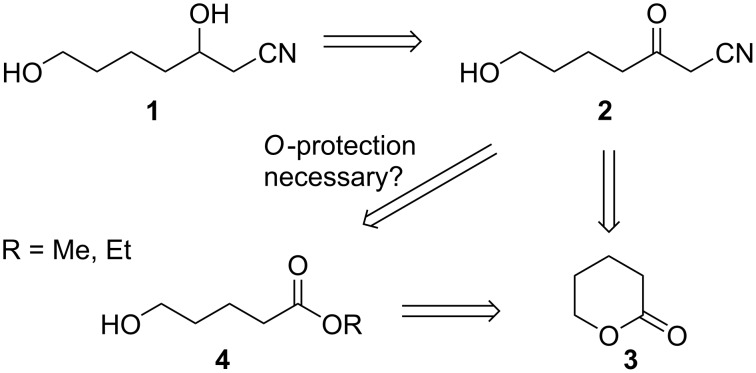
Proposed retrosynthesis of the free diol **1**.

We required a green, safe and scalable process for the facile production of *O*-unprotected hydroxylated β-hydroxynitrile **1** via trifunctionalized β-ketonitrile **2** by a direct, atom-economical ring opening of enolizable δ-valerolactone (**3**, Scheme 1). Although a two-step (or four-step, should hydroxy group *O*-protection prove to be necessary prior to acylation) protocol could, in theory, be envisaged to produce β-ketonitrile **2** from **3** by ring-opening esterification to afford hydroxy ester **4** followed by reaction with acetonitrile (CH_3_CN), a multistep approach would be less economical in industry (Scheme 1).

## Results and Discussion

**Scheme 2:**
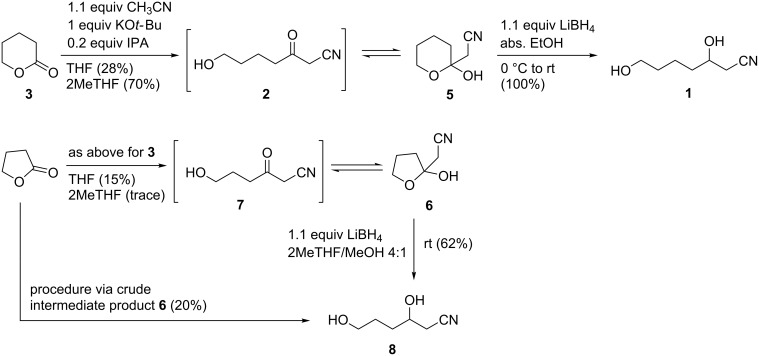
Preparation of *O*-unprotected, trifunctionalized synthons from lactones.

To date, the most economically appealing conditions from an environmentally friendly perspective entail relatively mild base-promoted (potassium *tert*-pentoxide or potassium *tert*-butoxide) acylation of substituted nitrile anions with esters under ambient conditions, as published by Ji et al. (2006) [9] and Kim et al. (2013) [10]. The large excess of ester and expensive base (potassium *tert*-pentoxide) required in the former method, in our opinion rendered it less economical and practical as a scalable option. Furthermore, when we applied Kim’s (KO*t*-Bu) method to δ-valerolactone, we obtained a highly viscous oil made up of a mixture of inseparable compounds that were unidentifiable by crude ^1^H NMR spectroscopy (see Supporting Information File 1). Similarly, using dry THF, 1 equiv CH_3_CN and 1 equiv KO*t*-Bu at rt, we obtained within minutes a precipitated amorphous gum which, upon work-up (partitioning between EtOAc and water) and removal of the solvents in vacuo, afforded a complex mixture of undesirable products by ^1^H NMR spectroscopy. However, to our delight, upon the addition of 20 mol % isopropanol (IPA) to the THF reaction mixture, we obtained a moderate amount of the desired product, albeit not as the open-chain β-ketonitrile **2**, but exclusively as the corresponding, cyclized hemiketal **5** (Scheme 2).

Furthermore, when using the green, sustainably produced solvent 2-methyltetrahydrofuran (2MeTHF) [11–12], in place of THF, we obtained a significantly higher yield of **5**. Remarkably, a crude yield of 80% of the hemiketal was obtained from technical grade, solidified δ-valerolactone that had previously been stored at rt (a compound which is known to polymerize unavoidably upon storage, and the technical grade reagent contains up to 25% polymerized material). The crude product was shown to be almost pure by ^1^H NMR and TLC. It can be seen in Supporting Information File 1 that the crude product obtained using 2MeTHF was significantly more enriched/purer in hemiketal **5** than that obtained using THF as the solvent. Pure product was readily obtained in 70% yield after purification by column chromatography. We reasoned that the lower aqueous miscibility of 2MeTHF, as compared to THF, resulted in a more efficient product extraction procedure, which further contributed to the increased yield of **5**. Varying the amount of IPA resulted in lower yields and more complex product mixtures. Substitution of IPA with *tert*-butanol or ethanol resulted in significantly lower product yields and a more complex product profile, as observed by crude product ^1^H NMR spectroscopy.

The mechanism by which IPA facilitates the reaction towards the formation of the cyclic hemiketal and indeed, the desired β-ketonitrile scaffold, has not been conclusively determined in this work. It is well known that strong-base deprotonation of acetonitrile leads to the formation of a nitrile-stabilized carbanion, in resonance with a ketene iminate anion. It has been calculated that the latter CN double-bonded species is relatively unstable compared to the CN triple-bonded carbanion species, which has the negative charge localized on the α-carbon atom [13]. This carbanion is therefore an excellent nucleophile and is usually generated from nitriles using metal amides or other strong bases [14–15]. Under our reaction conditions at rt, we propose that the presence of a catalytic amount of IPA increases the dielectric constant of the solvent as a whole, thereby increasing the solubility of both the KO*t*-Bu base and the acetonitrile derived KCH_2_CN salt. This effectively accelerates the generation of the latter and as such, its reaction with the lactone (or ester) carbonyl carbon. By increasing the solubility of the conjugate base salt, the p*K*_a_ of acetonitrile in the ethereal solvent is effectively lowered by adding a small amount of polar, protic IPA and this facilitates acetonitrile deprotonation. Alternatively, the addition of a crown ether is also known to enhance nucleophilic substitution reaction rates, but in this case through the suppression of ion-pairing [16]. We also investigated this option for the first time in the synthesis of β-ketonitriles from esters, as described later (see Table 1). Finally, it is probable that the presence of protic IPA suppresses enolization as the reaction progresses, by quenching KO*t*-Bu generated enolates and similarly, reactive alkoxide anions. This reduces the occurrence of undesirable side-reactions, e.g., intermolecular aldol reaction, lactone/ketonitrile product dimerization and ring-opening polymerization.

The application of this method to produce the analogous hemiketal **6** from γ-butyrolactone was not as efficient. Although the desired product **6** could indeed be isolated in 15% yield from γ-butyrolactone when the same reaction conditions were applied in THF, the use of 2MeTHF as solvent resulted in a complex mixture with only traces of the desired product, as revealed by crude product NMR spectroscopy. It is thought that the semivolatility of product **6** may have also resulted in lower yields compared to **5**.

It is well known that cyclic compounds often react differently dependent on their ring size, with 5-membered rings being generally more constrained and therefore more likely to ring-open than 6-membered rings. For example, in sugars, hemiacetal pyranoses (due to reduced bond angle strain) are energetically much more favored in solution compared to the corresponding 5-membered furanoses [17]. Significantly, ^1^H (500 MHz) and ^13^C NMR (126 MHz) of the purified product **6** (pure by TLC) indicated traces of what appears to be the uncyclized β-ketonitrile **7** (see Supporting Information File 1). This suggests that, in solution, the keto–hemiketal equilibrium for the butyrolactone product does not favor completely hemiketal **6**, as was observed for the 6-membered cycle **5**. The authors therefore propose that formation of the 6-membered hemiketal ring is more energetically favored under these reaction conditions, compared to the analogous 5-membered system. This effectively shields both the enolizable ketone and the sterically unhindered primary hydroxy functional groups in the valerolactone-derived product in situ, which reduces the occurrence of side-reactions and side-products, e.g., aldol reactions and polymerization, and results in a higher yield of the desired product compared to that obtained from γ-butyrolactone.

With compounds **5** and **6** in hand, simple and mild reduction protocols at rt satisfyingly afforded the desired diols **1** and **8**, thereby indicating that the hemiketals are still fully reducible under standard ketone reduction conditions. Purification of **6** afforded only a 10% overall yield of the diol **8**, but the direct conversion of crude intermediate product **6** resulted in a doubling of the overall yield of the diol from lactone to 20% (Scheme 2).

Due to the interest in the potential preparation of pyrimidines from β-ketonitriles and guanidine [18], we wanted to test the general applicability of this method for access to a variety of alkyl and aryl-substituted β-ketonitriles (as summarized in Table 1). When we applied this methodology to ethyl cyclopropane carboxylate in THF, we were delighted to obtain the desired (relatively volatile) product **9** in modest yield, because in our hands previous attempts to prepare **9** in sufficient amounts by following a literature procedure using NaH (in refluxing THF) [8] had failed. The reaction, again with 20 mol % IPA added and under an inert N_2_ atmosphere, afforded a similar product yield using 2MeTHF as solvent. Increasing the reaction time to 24 h did not increase the product yield, and residual starting material was observed throughout the reaction course, by TLC monitoring. Due to the persistent presence of unreacted starting materials in all examples given, it was decided to dose with second equivalents of base (KO*t*-Bu) and CH_3_CN after 1 h of reaction, as seen in most examples in Table 1. Disappointingly, we were unable to further significantly increase product yields, for example for the phenyl-substituted fluoxetine (Prozac^®^) intermediate **10** [19], which remained at ca. 50%.

**Table 1:** Preparation of a variety of β-ketonitriles.

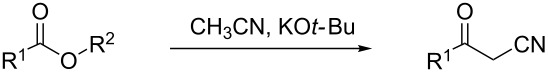								

Entry	Product	R^1^	R^2^	CH_3_CN (equiv)	KO*t*-Bu (equiv)	Additive (equiv)	Solvent (time at rt)	Yield (%)

1	**9**	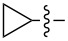	Et	2	2	IPA (0.2)	2MeTHF (2 h)	48^a^
2	**10**	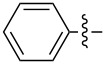	Me	2	2	IPA (0.2)	2MeTHF (2 h)	47
3	**11**	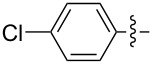	Me	2	2	IPA (0.2)	2MeTHF (1 h)	53
4	**12**	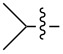	Me	2	2	IPA (0.2)	2MeTHF (2 h)	76^a^
5	**13**	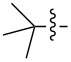	Me	2	2	IPA (0.2)	2MeTHF (2 h)	47
6	**14**	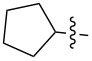	Me	2	2	IPA (0.2)	2MeTHF (2 h)	43
7	**15**	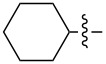	Me	2	2	IPA (0.2)	2MeTHF (2 h)	68
8	**16**	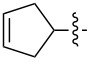	Me	2	2	IPA (0.2)	2MeTHF (2 h)	29^b^
9	**17**	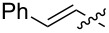	Et	1.5	1.1	IPA (0.2)	2MeTHF (30 min)	64
10	**18**	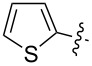	Me	1.2	1.2	IPA (0.1)	MTBE (2 h)	38
11	**18**	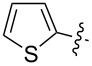	Me	1.2	1.2	18-crown-6 (0.1)	MTBE (30 min)	58
12	**18**	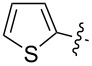	Me	1.2	1.2	18-crown-6 (0.1)	2MeTHF (30 min)	67

^a^Semivolatile products of which yield losses may have occurred upon solvent removal in vacuo. ^b^A significant amount of the corresponding carboxylic acid was isolated as side-product. This suggests that base-catalyzed ester alcoholysis may be a competing side-reaction.

Other methods were explored in an attempt to increase product yields. Although a putative radical mechanism that may be promoted in the presence of oxygen had previously been postulated by Kim et al. [10], we did not obtain increased product yields when carrying out the reaction in air. Furthermore, heating the reaction mixtures above rt when using NaH as a base in refluxing THF was clearly detrimental to product yield according to our previous observations and was not attempted.

Significantly, no side-products were observed in the organic extract after work-up, and a series of β-ketonitriles (some of which are semivolatile compounds) was successfully prepared and isolated under standard, ambient conditions in modest yields. The best yields were obtained for the 2-propyl **12** (76%) and cyclohexyl **15** (68%) analogues. We were pleased to obtain the highly versatile and synthetically useful cinnamoylacetonitrile **17** [20] from renewable ethyl cinnamate in 64% yield, as this yield was comparable to more expensive and hazardous lithiation protocols [7].

Finally, we attempted method optimization for the production of a commercially important pharmaceutical intermediate, thiophene-substituted β-ketonitrile precursor **18** of the antidepressant drug duloxetine (Cymbalta^®^) [21–22]. It was interesting to note that, although 38% of the thiophene product could be obtained using methyl *tert*-butyl ether (MTBE) with 0.1 equiv IPA as co-solvent/additive, a significantly higher yield of **18** was obtained when we used 10 mol % 18-crown-6 ether instead of IPA in a similarly catalytic amount. The yield was further improved to 67% using 2MeTHF as the solvent. It should be noted that the use of the crown ether resulted in recovery complications for some of the other compounds (e.g., when this methodology was applied to δ-valerolactone and γ-butyrolactone) and may therefore not be universally beneficial.

## Conclusion

In conclusion, mild, environmentally friendly procedures were developed for the preparation of trifunctionalized building blocks (hydroxylated β-ketonitriles) and valuable β-ketonitriles (including enolizable compounds) in modest to good yields from lactones and esters. We are currently investigating novel applications of diols **1** and **8**, as well as the application of this methodology for the synthesis of glycomimetic products from sugar lactones, and for the synthesis of various pyrimidines.

## Supporting Information

File 1 Experimental procedures, compound characterization data and NMR spectra.
