# Supplementary material for: A green, economical synthesis of β-ketonitriles and trifunctionalized building blocks from esters and lactones
Source: Beilstein J Org Chem. 2019 Dec 6;15:2930–5. doi: 10.3762/bjoc.15.287 (PMC6902896; doi:10.3762/bjoc.15.287)

BEILSTEIN JOURNAL OF ORGANIC CHEMISTRY

Supporting Information

for

A green, economical synthesis of β-ketonitriles and trifunctionalized building blocks from esters and lactones

Daniel P. Pienaar, Kamogelo R. Butsi, Amanda L. Rousseau and Dean Brady

Beilstein J. Org. Chem. **2019,** *15,* 2930–2935. doi:10.3762/bjoc.15.287

Experimental procedures, compound characterization data and NMR spectra

License and Terms: This is a supporting information file under the terms of the Creative Commons Attribution License (http://creativecommons.org/licenses/by/4.0). Please note that the reuse, redistribution and reproduction in particular requires that the authors and source are credited.
The license is subject to the *Beilstein Journal of Organic Chemistry* terms and conditions: (https://www.beilstein-journals.org/bjoc)

Table of Contents

General experimental information S2

Experimental procedures and compound characterization S3

References S9

NMR spectra S10

General experimental information:

Ethyl acetate (EtOAc) and *n*-hexane used for chromatographic techniques were both distilled prior to use by means of conventional distillation processes. Tetrahydrofuran (THF) was first dried over molecular sieves (4 Å), followed by distillation under inert nitrogen. Acetonitrile (CH_3CN) and dichloromethane (DCM) were distilled over calcium hydride, whereas THF was distilled over sodium with benzophenone as an indicator. 2-Methyltetrahydrofuran (2MeTHF) and methyl *tert*-butyl ether (MTBE) were pre-dried over molecular sieves (4 Å) but not distilled prior to use. All the required chemicals or reagents were obtained from Sigma–Aldrich or Merck, Acros Organics or Fluka, and were used without further purification.

Normal chromatography was performed on silica (SiO_2) gel 60 (Macherey-Nagel, particle size 0.063–0.200 mm), employing both isocratic and gradient eluent systems. Thin layer chromatography (TLC) of the compounds was executed either on Macherey-Nagel Alugram $_{SiO2}$ G/UV254 plates pre-coated with 0.25 mm silica gel 60 or on Merck TLC silica 60 F_{254} plates. The TLC plates were viewed under UV light (254 nm and 366 nm).

Nuclear magnetic resonance (NMR) spectra were recorded on either a Bruker AVANCE 300 MHz, or a Bruker AVANCE III 500 MHz spectrometer. All spectra were recorded in [D]chloroform ($CDCl_3$). Chemical shift values (*δ*) are reported in parts per million referenced against the signal of residual solvent ($CDCl_3$: δ_{1H} = 7.26 ppm, δ_{13C} = 77.16 ppm). Coupling constants (*J* values) are given in hertz (Hz).

Melting points were determined on a Reichert hot-stage microscope, and remain uncorrected. All crystalline compounds were recrystallized in the appropriate solvents prior to melting point determination.

High resolution mass spectra (HRMS) were obtained with a Bruker compact mass spectrometer. The sample was dissolved in methanol to a concentration of 1 ng/µL and introduced by direct infusion. The ionization mode was electrospray (ESI), positive ion mode with a capillary voltage of 4500 V and a desolvation temperature of 180 °C using nitrogen gas at 4.0 L/min.

Experimental procedures and compound characterisation

Synthesis of hemiketal **5**

Attempted synthesis of **5** using the method published by Kim et al. (see Results and Discussion in main text):

δ-Valerolactone (**3**, 75%, 2.87 g, 21.5 mmol) was dissolved with stirring for 5 min in technical-grade THF containing ca. 0.2% moisture (90 mL). Powdered KO*t*-Bu (95%, 5.08 g, 43 mmol) was added and the mixture was left stirring for 1 h under air. After adding MTBE (10 mL) and cooling on an ice bath, saturated NH_4Cl (50 mL) was added. The reaction was quenched by adding water (150 mL), followed by EtOAc (100 mL) and 12 M aq. HCl (3.2 mL). Layers were separated, the organic layer was washed with brine (50 mL), dried ($MgSO_4$) and the solvents evaporated in vacuo to afford a crude viscous oil product, the ^{1}H NMR spectrum of which is shown on p. 9.

Using 2MeTHF as solvent and 0.2 equiv IPA:

δ-Valerolactone (**3**, 75%, 2.30 g, 17.3 mmol) was dissolved with vigorous stirring in a mixture of 2MeTHF (60 mL), CH_3CN (1.35 mL, 25.8 mmol) and IPA (0.35 mL, 4.6 mmol) under a N_2 atmosphere. Powdered KO*t*-Bu (95%, 2.99 g, 25.3 mmol) was added over 1 min at rt and the mixture was left stirring for 30 min. After adding MTBE (10 mL) and cooling on an ice bath, saturated NH_4Cl (50 mL) was added. After warming to rt and stirring for another 5 min, the layers were separated in a separatory funnel. The organic phase was washed with brine (50 mL). The combined aqueous layer was extracted again with MTBE (2 × 50 mL). The combined organic layer was dried ($MgSO_4$), filtered and evaporated to yield 1.95 g (80%) of nearly pure hemiketal **5** by ^{1}H NMR spectroscopy. Subsequent SiO_2 gel column chromatography (20% EtOAc/hexane) afforded, after evaporation of solvents in vacuo by roto-evaporation, **5** (1.70 g, 12.0 mmol, 70%) as

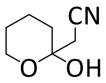

colorless oil. **R$_f$** = 0.56 (50% EtOAc/hexane); **^{1}H NMR** (300 MHz, $CDCl_3$) δ 3.99 – 3.84 (m, 1H), 3.79 – 3.66 (m, 1H), 2.85 (s, 1H), 2.66 (s, 2H), 1.96 – 1.74 (m, 2H), 1.75 – 1.50 (m, 3H); **^{13}C NMR** (75 MHz, $CDCl_3$) δ 116.66, 94.19, 62.19, 33.13, 32.80, 24.68, 18.65; **HRMS** (ES$^+$) $[M + H]^+$: 142.0863, found 142.840, $[M + Na]^+$: 164.0682, found 164.0654.

Using THF as solvent with 0.2 equiv IPA added :

Starting from 3.8 g δ-valerolactone (**3**, 75%, 28.5 mmol), the procedure was carried out exactly as set out above, except that THF was used as the solvent. The ^{1}H NMR spectrum of the crude product obtained

here is shown below in this document for comparison to that obtained by using 2MeTHF as solvent. After column chromatography as before, pure hemiketal **5** (1.12 g, 28%) was obtained as colorless yellow oil.

<u>Synthesis of hemiketal **6**</u>

The same procedure as described for hemiketal **5** was followed, except THF was used as the solvent. Starting from γ-butyrolactone (5.1 g, 59.2 mmol), CH_3CN (3.11 mL, 59.5 mmol), IPA (1.0 mL, 13 mmol) and KO*t*-Bu (95%, 7.0 g, 59.2 mmol), after SiO_2 gel column chromatography (20–50% EtOAc/hexane) and evaporation of the solvents in vacuo, **6** was obtained as colorless oil (1.1 g, 15%). $\mathbf{R_f}$ = 0.30 (50%

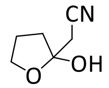

EtOAc/hexane); **^{1}H NMR** (500 MHz, $CDCl_3$) δ 4.11 (td, *J* = 8.2, 7.7, 4.0 Hz, 1H), 3.98 (td, *J* = 8.2, 7.6, 5.0 Hz, 1H), 2.88 – 2.66 (m, 3H), 2.24 – 2.09 (m, 2H), 2.08 – 1.96 (m, 2H); **^{13}C NMR** (126 MHz, $CDCl_3$) δ 116.73, 103.33, 69.34, 36.76, 30.00, 24.77; $[M + Na]^+$: 150.0526, found 150.0507.

<u>3,7-dihydroxyheptanenitrile **1**</u>

Hemiketal **5** (0.89 g, 6.3 mmol) was dissolved in abs. EtOH (25 mL) under N_2 and cooled to 0 °C on an ice-bath. $LiBH_4$ (151 mg, 6.93 mmol) was added, the mixture was gradually warmed to rt and stirred for 4 h. After cooling on an ice-bath, sat. NH_4Cl (20 mL) and EtOAc (20 mL) were added. After warming to rt, the

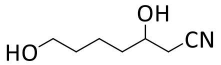

layers were separated and the organic layer was washed with brine (10 mL), dried ($MgSO_4$) and solvents evaporated in vacuo to yield crude diol product, which was purified by SiO_2 gel chromatography (EtOAc) to yield **1** (898 mg, 100%) as viscous, colorless oil. $\mathbf{R_f}$ = 0.10 (75% EtOAc/hexane); **^{1}H NMR** (300 MHz, $CDCl_3$) δ 4.23 – 3.97 (m, 1H), 3.97 – 3.83 (m, 1H), 3.71 – 3.49 (m, 2H), 2.62 – 2.38 (m, 2H), 1.75 – 1.34 (m, 7H); **^{13}C NMR** (75 MHz, $CDCl_3$) δ 118.15, 67.34, 62.20, 36.09, 31.91, 26.22, 21.75; **HRMS** (ES^+) $[M + H]^+$: 144.1020, found 144.1018, $[M + Na]^+$: 166.0839, found 166.0833.

<u>3,6-dihydroxyhexanenitrile **8**</u>

<u>Procedure A:</u> The same procedure as described for diol **1** was followed from hemiketal **6** (0.31 g, 2.44 mmol) in 2MeTHF (8 mL) and MeOH (2 mL) to afford, after SiO_2 gel chromatography (EtOAc), **8**

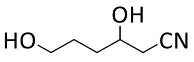

(195 mg, 62%) as viscous, colorless oil. $\mathbf{R_f}$ = 0.25 (EtOAc); **^{1}H NMR** (300 MHz, Chloroform-*d*) δ 4.16 – 3.90 (m, 2H), 3.82 – 3.64 (m, 2H), 2.65 – 2.38 (m, 3H), 1.87 – 1.61 (m, 4H).

^{13}**C NMR** (75 MHz, $CDCl_3$) δ 117.99, 67.71, 62.70, 34.28, 28.60, 26.26; **HRMS** (ES^+) $[M + H]^+$: 130.0863, found 130.0839, $[M + Na]^+$: 152.0682, found 152.0656.

Procedure B: γ-Butyrolactone (**3**, 2.0 g, 23.2 mmol) was dissolved in a mixture of THF (80 mL), CH_3CN (1.45 mL, 27.8 mmol) and IPA (0.356 mL, 4.66 mmol), under a N_2 atmosphere. Powdered KO*t*-Bu (95%, 3.12 g, 26.4 mmol) was added over 1 min at rt and the mixture was left stirring for 30 min. After adding MTBE (10 mL) and cooling on an ice bath, saturated NH_4Cl (50 mL) was added. After warming to rt and stirring for another 5 min, the layers were separated in a separatory funnel. The organic phase was washed with brine (50 mL). The combined aqueous layer was extracted with DCM (2 × 50 mL). The combined organic layer was dried ($MgSO_4$), filtered and evaporated to yield 1.39 g of crude hemiketal **6**, which was dissolved in 2MeTHF (32 mL) and MeOH (8 mL) and cooled to 0 °C on an ice-bath. $LiBH_4$ (0.26 g, 11.9 mmol) was added, the mixture was warmed to rt and stirred for 18 h. After work-up (see procedure for diol **1** above), and purification as in Procedure A, diol **8** (591 mg, 20% over 2 steps) was obtained.

Synthesis of methyl thiopene-2-carboxylate

To a solution of 2-thiophenecarboxylic acid (2.0 g, 15.6 mmol) in DMSO (30 mL) under N_2 was added NaO*t*-Bu (1.8 g, 18.7 mmol) and the mixture was stirred for 10 min. After cooling to 0 °C on an ice-bath, iodomethane (1.5 mL, 24.1 mmol) was added dropwise over 1 min. The mixture was warmed to rt and stirred 18 h. After cooling to 0 °C, H_2O (50 mL) and MTBE (100 mL) were added. The layers were separated and the organic layer was washed with saturated $NaHCO_3$ (3 × 70 mL), dried ($MgSO_4$) and evaporated in vacuo to yield crude product which was purified by SiO_2 gel column chromatography (5% EtOAc/hexane) to yield methyl thiophene-2-carboxylate (2.16 g, 97%).

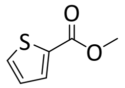

R$_f$ = 0.49 (15% EtOAc/hexane); ^{1}**H NMR** (300 MHz, $CDCl_3$) δ 7.79 (dd, *J* = 3.7, 1.3 Hz, 1H), 7.54 (dd, *J* = 5.0, 1.3 Hz, 1H), 7.09 (dd, *J* = 5.0, 3.8 Hz, 1H), 3.88 (s, 3H); ^{13}**C NMR** (75 MHz, $CDCl_3$) δ 162.79, 133.70, 133.56, 132.43, 127.84, 52.24; **HRMS** (ES^+) $[M + H]^+$: 143.0162, found 143.0139, $[M + Na]^+$: 164.9981, found 164.9955. [1]

General procedure for the synthesis of the β-ketonitriles

To a solution of methyl/ethyl ester (1.0 equiv) in 20 mL 2-MeTHF under N_2 was added IPA (0.2 equiv), CH_3CN (1.0 equiv), followed by KO*t*-Bu (1.0 equiv). The reaction mixture was stirred at room temperature for an hour under N_2 atmosphere, then dosed-up with an additional 1.0 mol equiv of CH_3CN and KO*t*-Bu, respectively. The reaction mixture was stirred for another hour, quenched with 200 mL of saturated NH_4Cl

solution and the layers separated. The aqueous layer was extracted with 200 mL DCM. The combined organic layer was dried over Na_2SO_4, filtered and the solvents removed in vacuo by roto-evaporation. The resulting residue was purified by SiO_2 gel column chromatography using EtOAc/hexane mixtures as mobile phases.

3-Cyclopropyl-3-oxopropanenitrile (**9**)

A mixture of ethyl cyclopropane carboxylate (2.00 mL, 16.8 mmol), CH_3CN (1.75 mL, 33.6 mmol), KO*t*-Bu (3.77 g, 33.6 mmol) and IPA (0.26 mL, 3.4 mmol) in 2-MeTHF afforded **9** as light yellow oil (0.90 g, 48%).

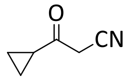

R_f = 0.56 (1:1 EtOAc/Hex); **^{1}H NMR** (300 MHz, $CDCl_3$) δ 3.65 (s, 2H), 2.14 – 1.90 (m, 1H), 1.22 – 0.85 (m, 4H); **^{13}C NMR** (75 MHz, $CDCl_3$) δ 198.43, 114.50, 33.02, 20.55, 13.01; **HRMS** (ES^+) $[M + H]^+$: 110.0606, found 110.0590, $[M + Na]^+$: 132.0420, found 132.0408.

3-Oxo-3-phenylpropanenitrile (**10**)

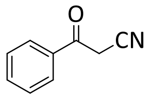

A mixture of methyl benzoate (4.00 mL, 31.7 mmol), CH_3CN (3.40 mL, 63.4 mmol), IPA (0.48 mL, 6.3 mmol) and KO*t*-Bu (7.11 g, 63.4 mmol) in 2-MeTHF (40 mL) afforded 3-oxo-phenylpropanenitrile (**10**) as light yellow solid (2.18 g, 47%). **R_f** = 0.68 (1:1 EtOAc/Hex); **m.p.** °C (lit. m.p. 120-122 °C) [2]; **^{1}H NMR** (300 MHz, $CDCl_3$) δ 7.98 – 7.87 (m, 2H), 7.73 – 7.62 (m, 1H), 7.59 – 7.47 (m, 2H), 4.10 (s, 2H); **^{13}C NMR** (75 MHz, $CDCl_3$) δ 187.26, 134.85, 134.38, 129.27, 128.58, 113.94, 29.54; **HRMS** (ES^+) $[M + Na]^+$: 168.0420, found 168.0403. [2]

3-Oxo-3-(4-chlorophenyl)propanenitrile (**11**)

A mixture of methyl 4-chlorobenzoate (1.20 g, 7.03 mmol), CH_3CN (0.735 mL, 14.1 mmol), IPA (0.108 mL, 1.41 mmol) and KO*t*-Bu (1.58 g, 14.1 mmol) in 2-MeTHF (30 mL), after a reaction time of 1 h in total at

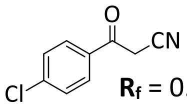

rt, afforded 3-oxo-3(4-chlorophenyl)propanenitrile (**11**) as white solid (0.673 g, 53%). **R_f** = 0.52 (30% EtOAc/Hex); **m.p.** 125-127 °C (lit. m.p. 124-126 °C) [2]; **^{1}H NMR** (400 MHz, $CDCl_3$) δ 7.91 – 7.83 (m, 2H), 7.55 – 7.47 (m, 2H), 4.05 (s, 2H); **^{13}C NMR** (101 MHz, $CDCl_3$) δ 186.05, 141.66, 132.71, 129.99, 129.72, 113.54, 29.52; **HRMS** (ES^+) $[M + H]^+$: 180.0211, found 180.0491, $[M + Na]^+$: 202.0030, found 202.0335. [2]

4-Methyl-3-oxopentanenitrile (**12**)

A mixture of methyl isobutyrate (2.00 mL, 17.4 mmol), 2-MeTHF (20 mL), IPA (0.27 mL, 3.48 mmol), CH_3CN (1.80 mL, 34.8 mmol) and KO*t*-Bu (3.92 g, 34.8 mmol) afforded **12** as yellow oil (1.46 g, 76%).

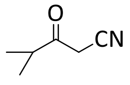

R_f = 0.68 (1:1 EtOAc/Hex); **^{1}H NMR** (300 MHz, $CDCl_3$) δ 3.56 (s, 2H), 2.71 (hept, *J* = 6.9 Hz, 1H), 1.09 (d, *J* = 7.0 Hz, 6H); **^{13}C NMR** (75 MHz, $CDCl_3$) δ 201.69, 114.18, 40.46, 30.13, 17.70 **HRMS** (ES^+) $[M + H]^+$: 112.0762, found 112.0750, $[M + Na]^+$: 134.0577, found 134.0568. [3]

4,4-Dimethyl-3-oxopentanenitrile (**13**)

A mixture of methyl pivalate (2.00 mL, 15.0 mmol), CH_3CN (1.57 mL, 30.0 mmol), KO*t*-Bu (3.37 g, 30.0 mmol) and IPA (0.23 mL, 3.0 mmol) afforded compound **13** as light yellow solid (0.89 g, 47%).

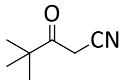

R_f = 0.78 (1:1 EtOAc/Hex); **m.p.**: 68 °C (lit m.p. 67-68 °C) [2]; **^{1}H NMR** (300 MHz, $CDCl_3$) δ 3.63 (s, 2H), 1.19 (s, 9H); **^{13}C NMR** (75 MHz, $CDCl_3$) δ 202.77, 114.16, 44.74, 27.53, 26.22; **HRMS** (ES^+) $[M + H]^+$: 126.0919, found 126.0906, $[M + Na]^+$: 148.0733, found 148.0724. [2]

3-Cyclopentyl-3-oxopropanenitrile (**14**)

A mixture of methyl cyclopentane carboxylate (2.42 g, 15.6 mmol), CH_3CN (1.63 mL, 31.2 mmol), KO*t*-Bu (3.80 g, 31.2 mmol) and IPA (0.24 mL, 3.1 mmol) afforded **14** as yellow oil (0.92 g, 43%).

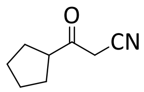

R_f = 0.94 (1:1 EtOAc/Hex); **^{1}H NMR** (300 MHz, Chloroform-*d*) δ 3.53 (s, 2H), 3.00 (tt, *J* = 8.8, 7.0 Hz, 1H), 1.98 – 1.47 (m, 8H); **^{13}C NMR** (75 MHz, $CDCl_3$) δ 200.15, 114.18, 50.83, 31.23, 28.78, 25.88; **HRMS** (ES^+) $[M + Na]^+$: 160.0733, found 160.0710. [4]

3-Cyclohexyl-3-oxopropanenitrile (**15**)

A mixture of methyl cyclohexane carboxylate (2.00 mL, 14.0 mmol), CH_3CN (1.46 mL, 28.0 mmol), KO*t*-Bu (3.14 g, 28.0 mmol) and IPA (0.21 mL, 2.8 mmol) afforded **15** as yellow oil (1.43 g, 68%).

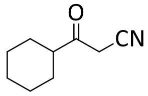

R_f = 0.93 (1:1 EtOAc/Hex); **^{1}H NMR** (300 MHz, $CDCl_3$) δ 3.53 (s, 2H), 2.48 (ddq, *J* = 11.0, 7.0, 3.4 Hz, 1H), 2.04 – 1.00 (m, 10H); **^{13}C NMR** (75 MHz, $CDCl_3$) δ 200.74, 114.17, 49.98, 30.38, 28.10, 25.49, 25.22 **HRMS** (ES^+) $[M + Na]^+$: 174.0890, found 174.0866. [5]

3-(Cyclopent-3-en-1-yl)-3-oxopropanenitrile (**16**)

A mixture methyl 3-cyclopentenecarboxylate (0.80 mL, 6.5 mmol), CH_3CN (0.68 mL, 13.0 mmol), KO*t*-Bu (1.46 g, 13.0 mmol) and IPA (0.10, 1.3 mmol) afforded **16** as brown oil (0.39 g, 29%).

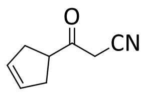

R_f = 0.61 (1:1 EtOAc/Hex); **^{1}H NMR** (300 MHz, $CDCl_3$) δ 5.59 (s, 2H), 3.57 (s, 2H), 3.35 (ddd, *J* = 15.9, 8.9, 7.0 Hz, 1H), 2.70 – 2.47 (m, 4H); **^{13}C NMR** (75 MHz, $CDCl_3$) δ 199.14, 128.48, 114.12, 48.41, 34.86, 30.72; **HRMS** (ES^+) $[M + H]^+$: 136.0757, found 136.0746.

Cinnamoyl acetonitrile (**17**)

The general procedure was followed without dosing second equivalents of CH_3CN and KO*t*-Bu. Thus, a mixture of ethyl cinnamate (0.90 g, 5.1 mmol), CH_3CN (0.32 mL, 6.1 mmol), IPA (0.078 mL, 1.0 mmol) and KO*t*-Bu (0.69 g, 5.8 mmol) in 2MeTHF (30 mL) afforded, after 30 min reaction at rt, and work-up, **17** (0.56 g, 64%) as light yellow powder. **R_f** = 0.41 (30% EtOAc/Hex); **m.p.** 121-122 °C (lit. m.p. 125-127 °C) [6]; **^{1}H NMR** (300 MHz, $CDCl_3$) δ 7.68 (d, *J* = 16.0 Hz, 1H), 7.62 – 7.55 (m, 2H), 7.50 – 7.39 (m, 3H), 6.87 (d, *J* = 16.0 Hz, 1H), 3.72 (s, 2H); **^{13}C NMR** (75 MHz, $CDCl_3$) δ 186.44, 146.73, 133.51, 131.83, 129.32, 128.98, 122.50, 114.13, 30.98; **HRMS** (ES^+) $[M + H]^+$: 172.0757, found 172.0732, $[M + Na]^+$: 194.0577, found 194.0551. [6]

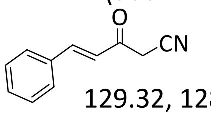

3-Oxo-3-(thiophen-2-yl)propanenitrile (**18**)

Procedure A: The general procedure was followed without dosing second equivalents of CH_3CN and KO*t*-Bu. Thus, a mixture of methyl thiophene-2-carboxylate (0.80 g, 5.63 mmol), CH_3CN (0.35 mL, 6.7 mmol), IPA (0.050 mL, 0.65 mmol) and KO*t*-Bu (95%, 0.80 g, 6.8 mmol) in MTBE (50 mL) afforded, after 30 min reaction at rt and work-up, **18** (0.327 g, 38%) as a recrystallized (5% EtOAc/hexane) yellow solid. **R_f** = 0.30 (30% EtOAc/Hex); **m.p.** 133-134 °C (lit. m.p. 131-133 °C) [7]; **^{1}H NMR** (300 MHz, $CDCl_3$) δ 7.84 – 7.74 (m, 2H), 7.20 (dd, *J* = 4.9, 3.9 Hz, 1H), 4.01 (s, 2H); **^{13}C NMR** (75 MHz, $CDCl_3$) δ 179.67, 141.01, 136.38, 133.84, 128.85, 113.58, 29.70; **HRMS** (ES^+) $[M + H]^+$: 152.0165, found 152.0149, $[M + Na]^+$: 173.9985, found 173.9968. [7]

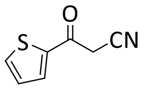

Procedure B: As described in Procedure B, however, starting from methyl thiophene-2-carboxylate (1.5 g, 10.5 mmol), and adding 18-crown-6 ether (0.33 g, 0.125 mmol) in place of IPA, in MTBE (50 mL), afforded **18** (0.929 g, 58%) as a recrystallized yellow solid.

Procedure C: As described in Procedure B, however, starting from methyl thiophene-2-carboxylate (0.708 g, 4.96 mmol), and adding 18-crown-6 ether (0.15 g, 0.57 mmol), in 2MeTHF (24 mL), afforded **18** (0.513 g, 67%) as recrystallized yellow solid.

References

1. Y. Zhu, H. Yan, L. Lu, D. Liu, G. Rong, J. Mao, J. Org. Chem. 78 (2013) 9898-9905.
2. J. Sun, H. Ge, X. Zhen, X. An, G. Zhang, D. Zhang-Negrerie, Y. Du, K. Zhao, Tetrahedron 74 (2018) 2107-2114.
3. Y. Chen, S. M. Sieburth, Synthesis 15 (2002) 2191-2194.
4. M. W. Rowbottom, R. Faraoni, Q. Chao, B. T. Campbell, A. G. Lai, E. Setti *et al.*, J. Med. Chem. 55 (2012) 1082-1105.
5. H. Shen, J. Li, Q. Liu, J. Pan, R. Huang,Y. Xiong, J. Org. Chem. 80 (2015) 7212-7218.
6. M. Rehan, R. Nallagonda, B. G. Das, T. Meena, P. Ghorai, J. Org. Chem. 82 (2017) 3411-3424.
7. A. L. Cardoso, L. Gimeno, A. Lemos, F. Palacios, T. M. V. D. Pinho e Melo, J. Org. Chem. 78 (2013) 6983-6991.

Crude ^{1}H NMR for attempted preparation of 5 using the method by Kim et al. ($CDCl_3$, 300 MHz)

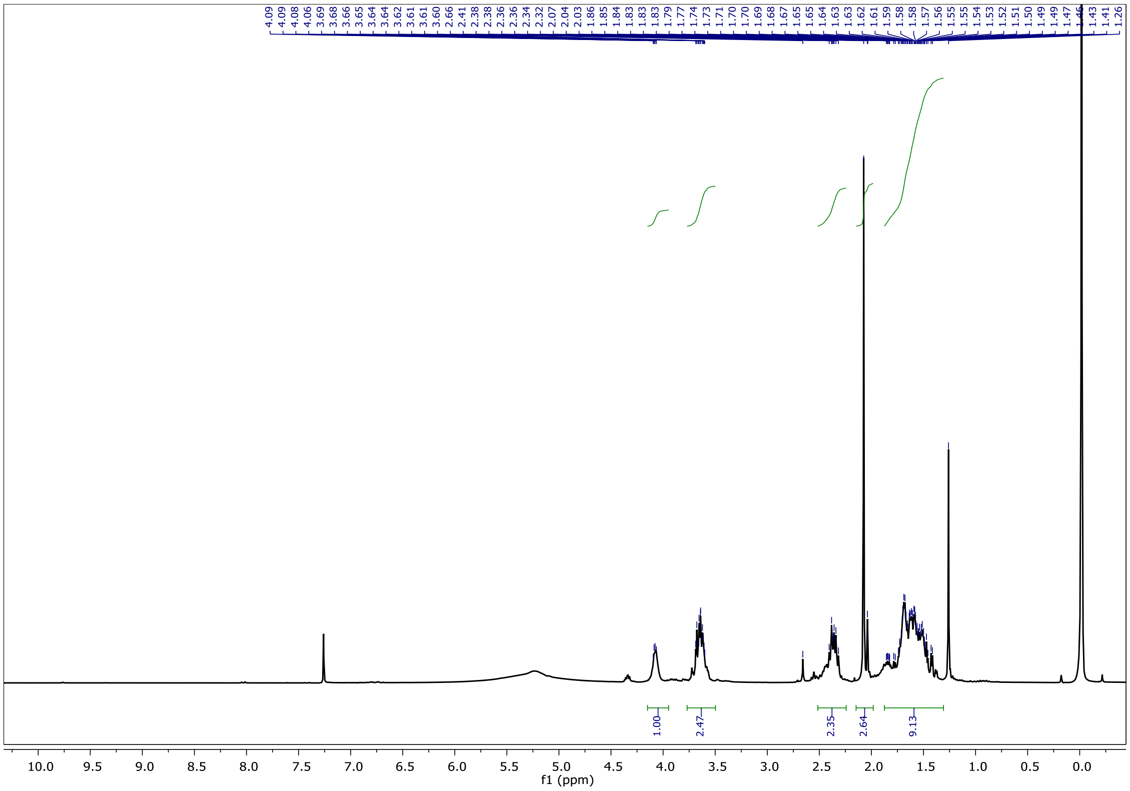

Crude ^{1}H NMR for the preparation of 5 using our THF–IPA method ($CDCl_3$, 300 MHz)

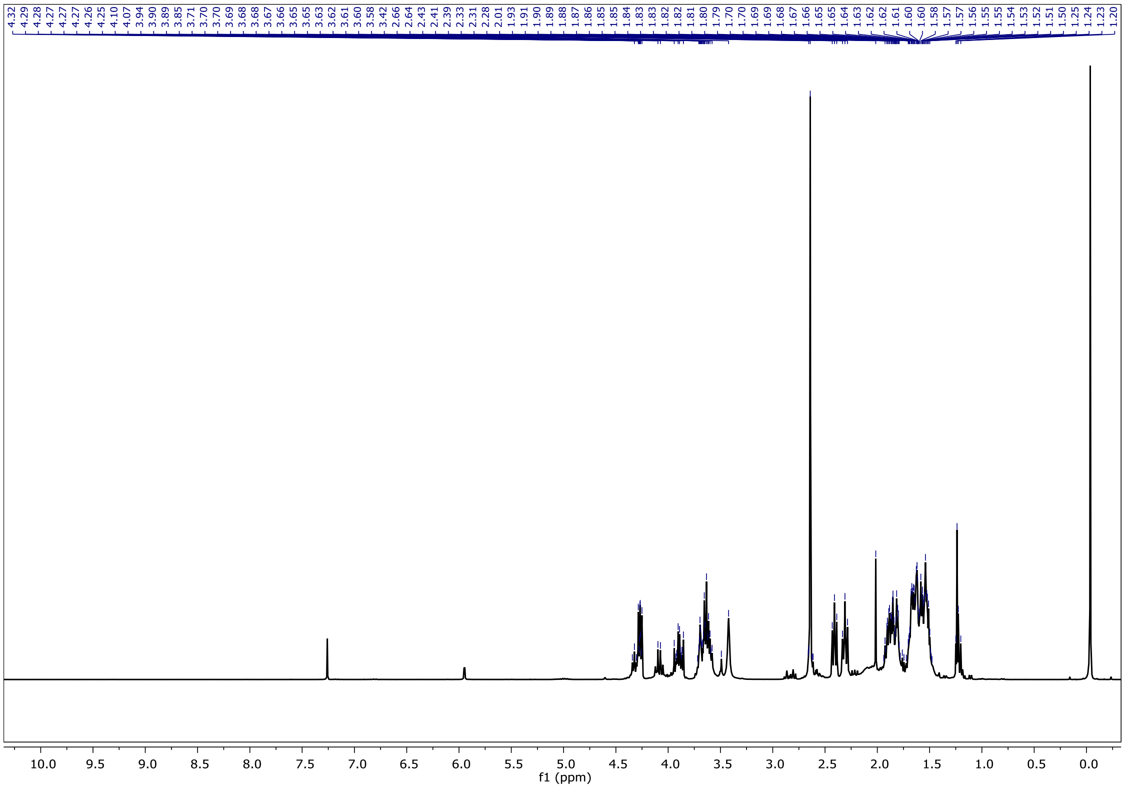

Crude ^{1}H NMR for the preparation of 5 using our 2MeTHF–IPA method ($CDCl_3$, 300 MHz)

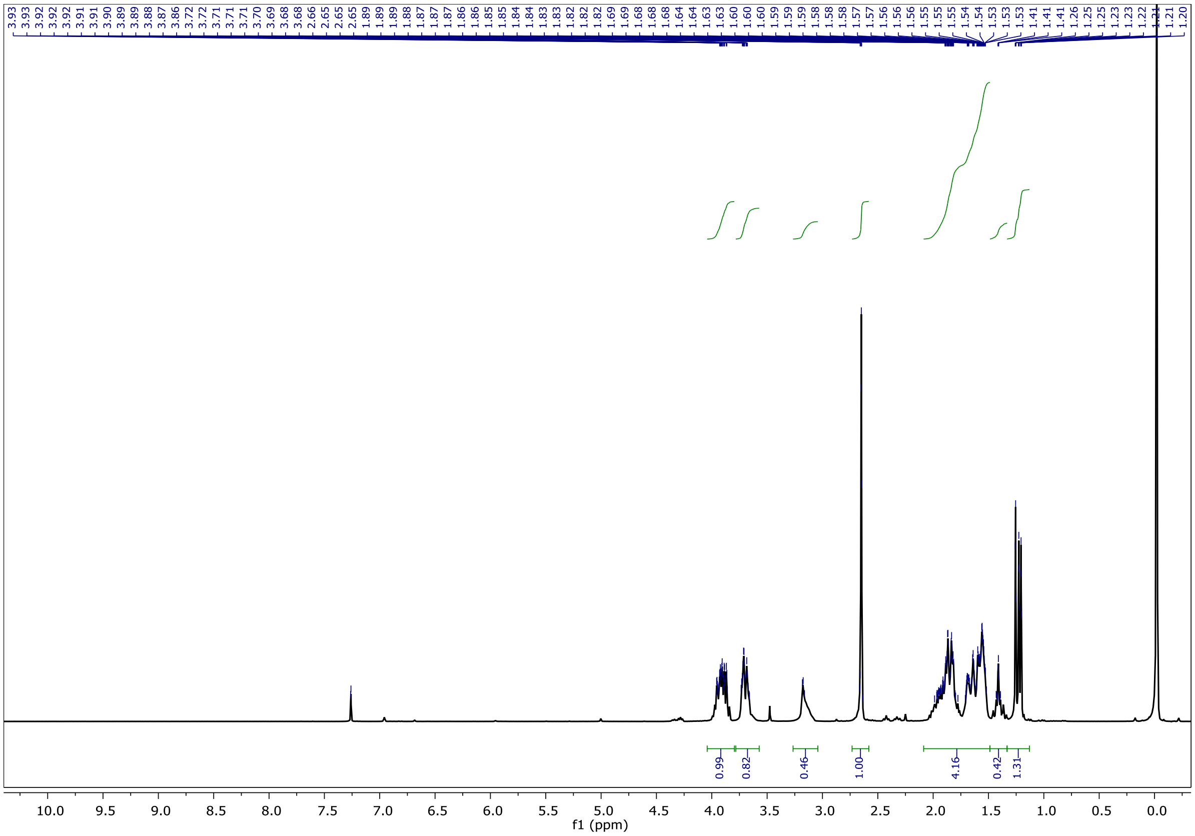

^{1}H NMR for pure 5 ($CDCl_3$, 300 MHz)

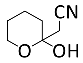

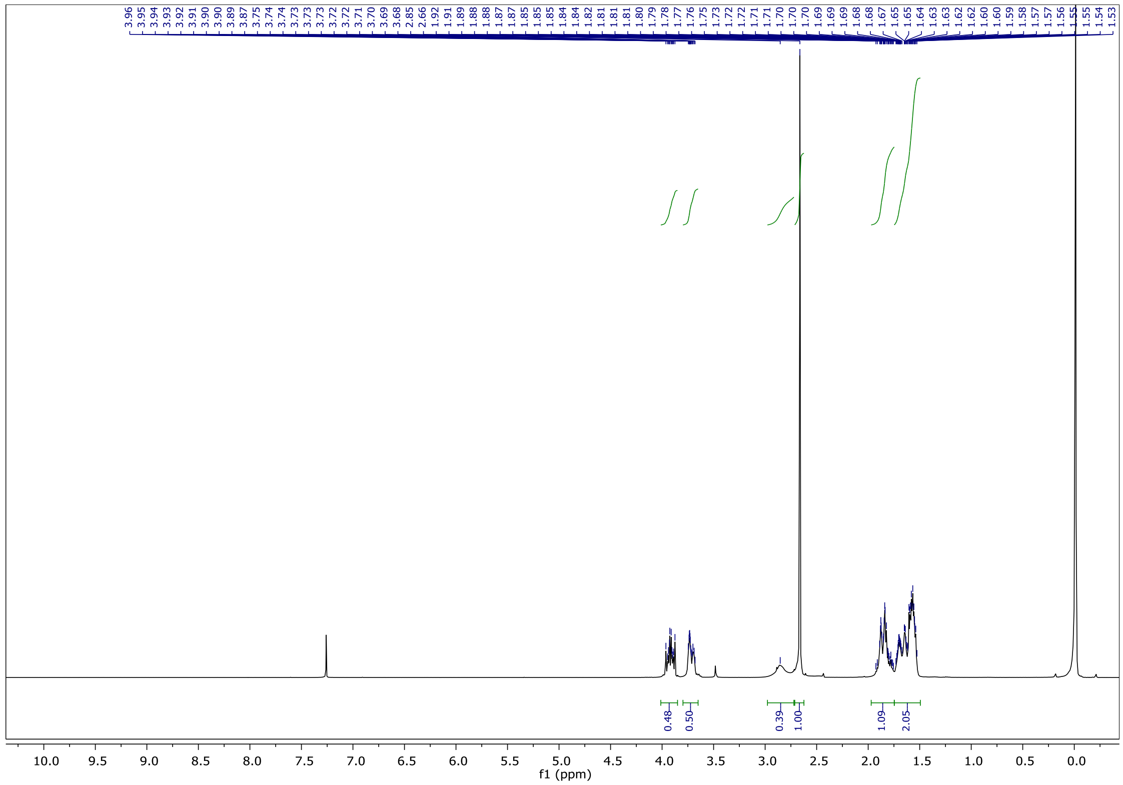

^{13}C NMR for 5 ($CDCl_3$, 75 MHz)

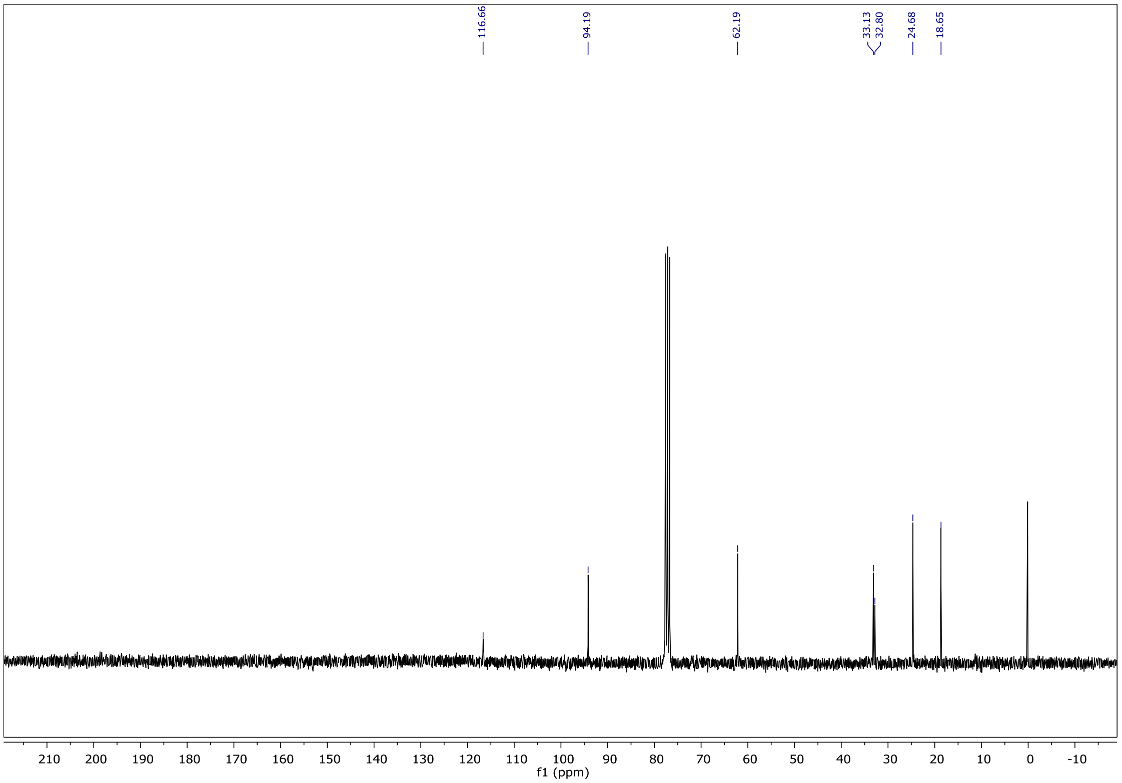

^{1}H NMR for 1 ($CDCl_3$, 300 MHz)

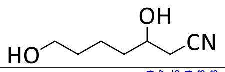

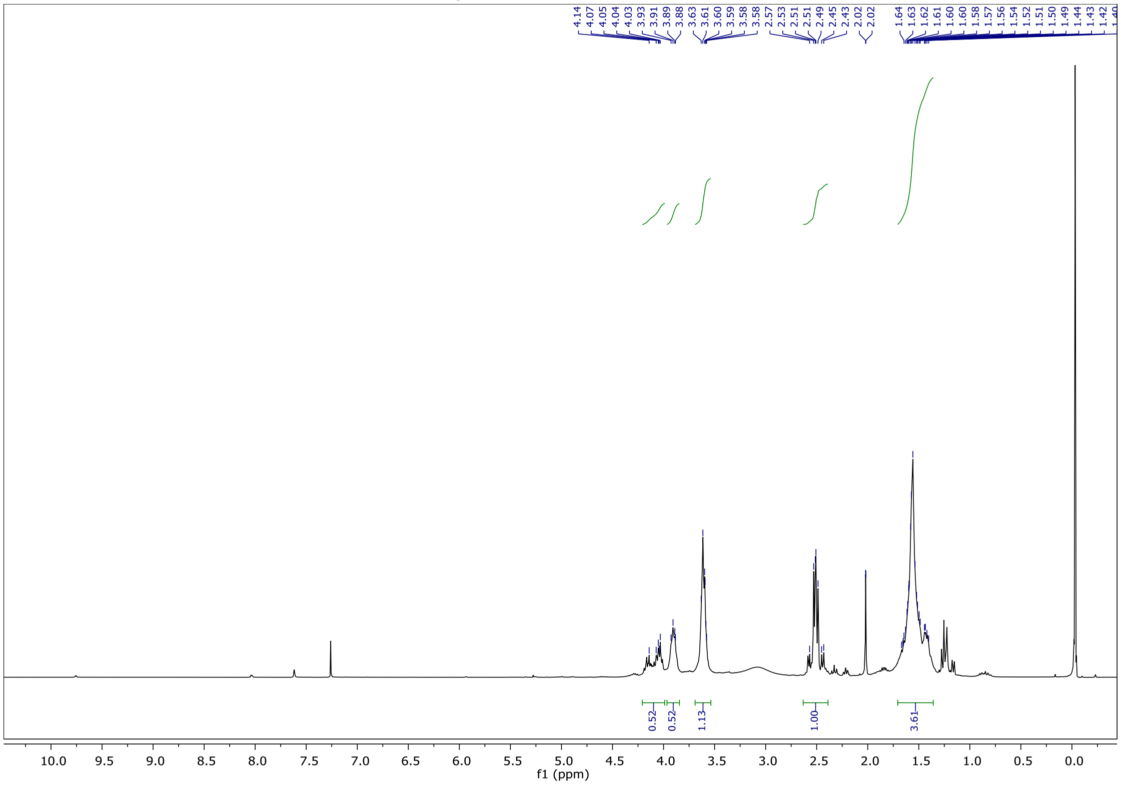

^{13}C NMR for 1 ($CDCl_3$, 75 MHz)

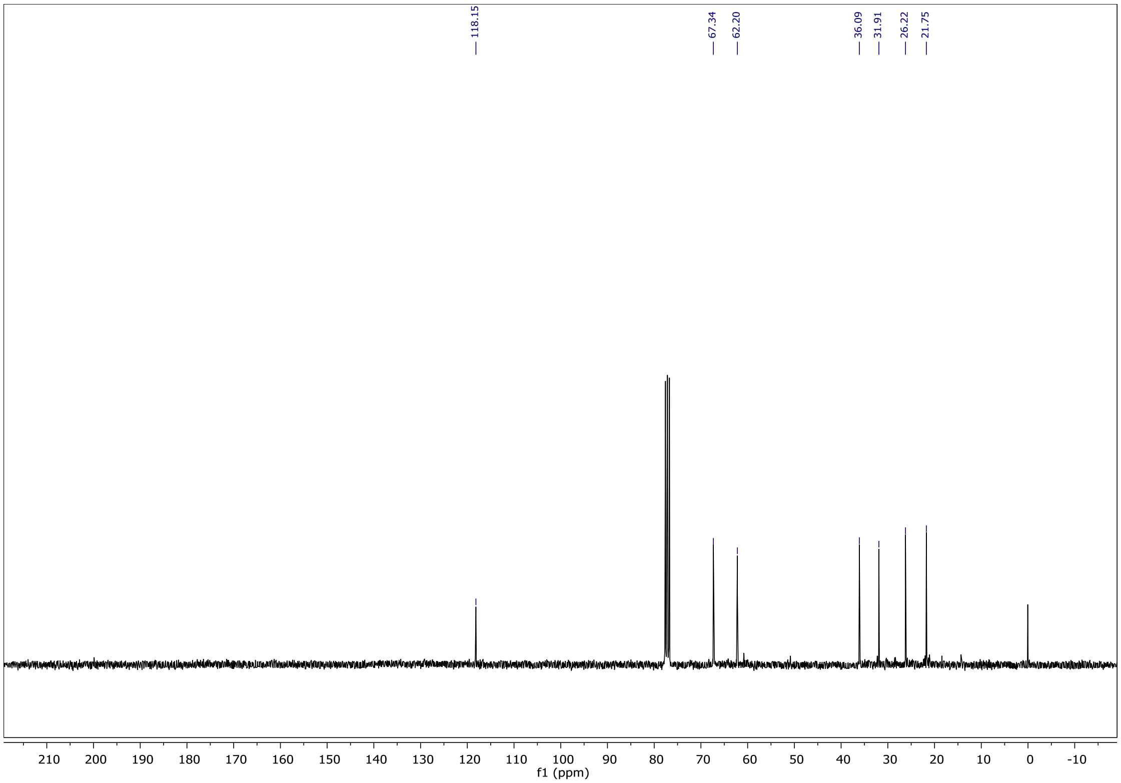

^{1}H NMR for 6 (CDCl$_3$, 500 MHz) 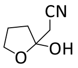

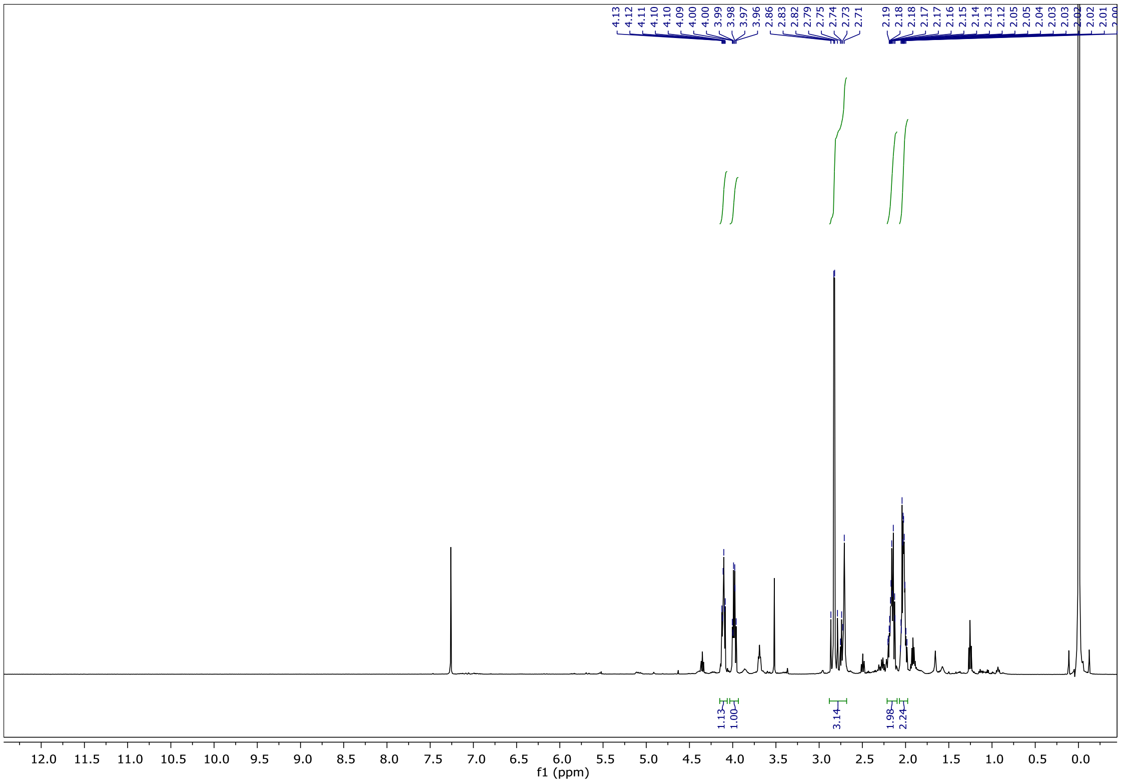

^{13}C NMR for 6 (CDCl$_3$, 126 MHz)

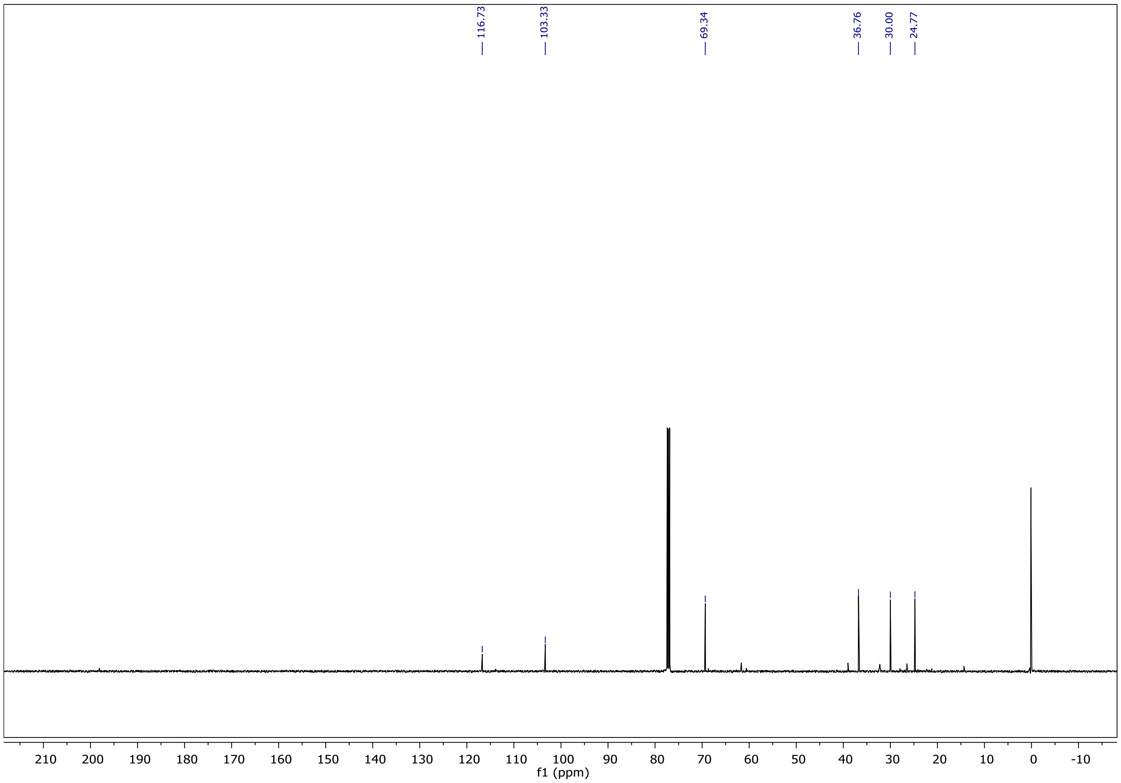

^{1}H NMR for 8 ($CDCl_3$, 300 MHz)

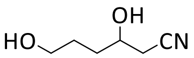

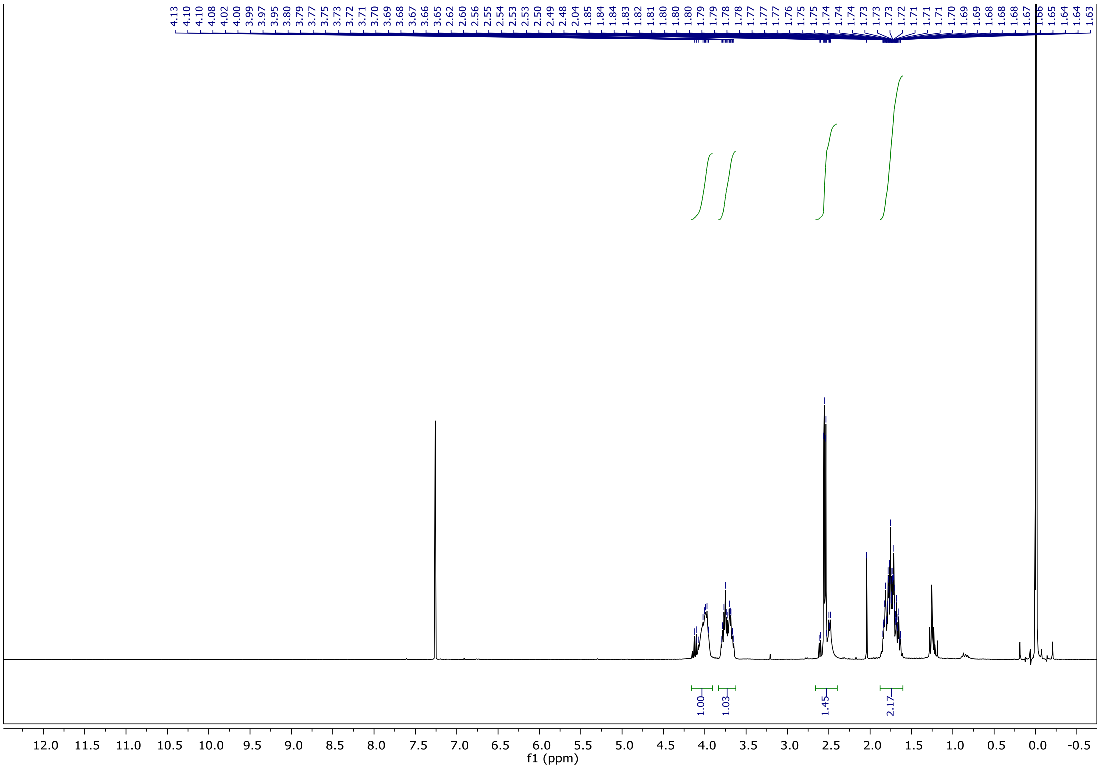

^{13}C NMR for 8 ($CDCl_3$, 75 MHz)

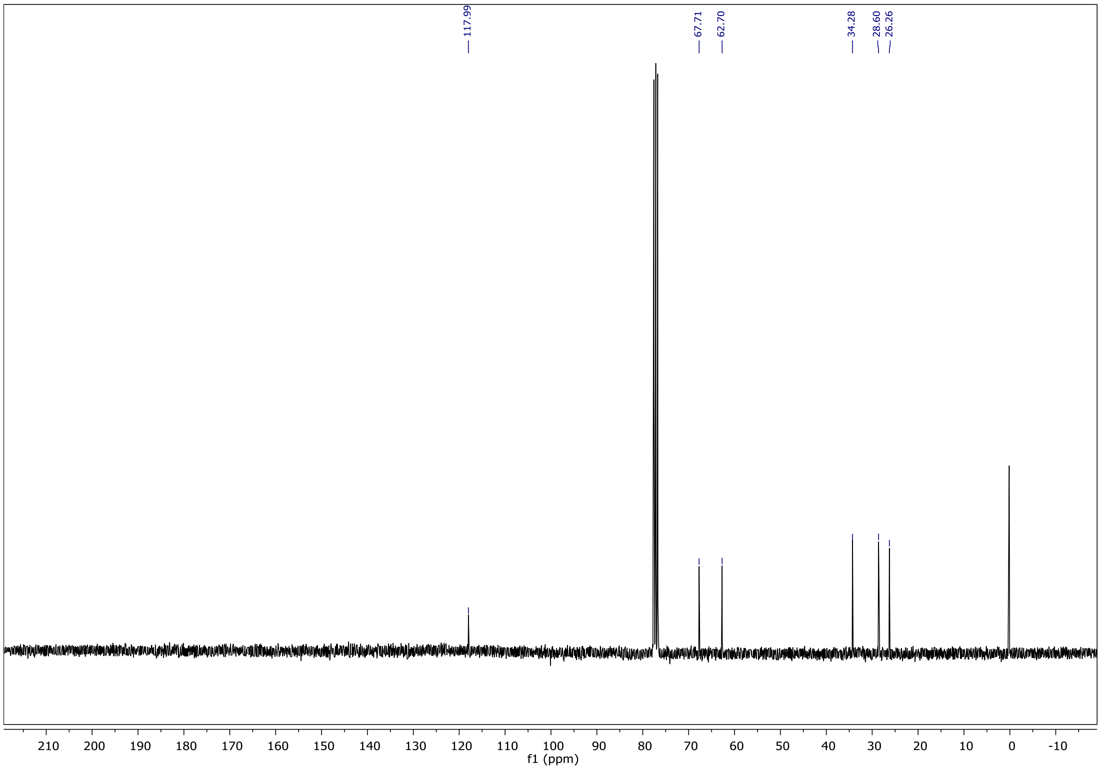

^{1}H NMR for 9 ($CDCl_3$, 300 MHz)

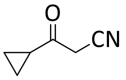

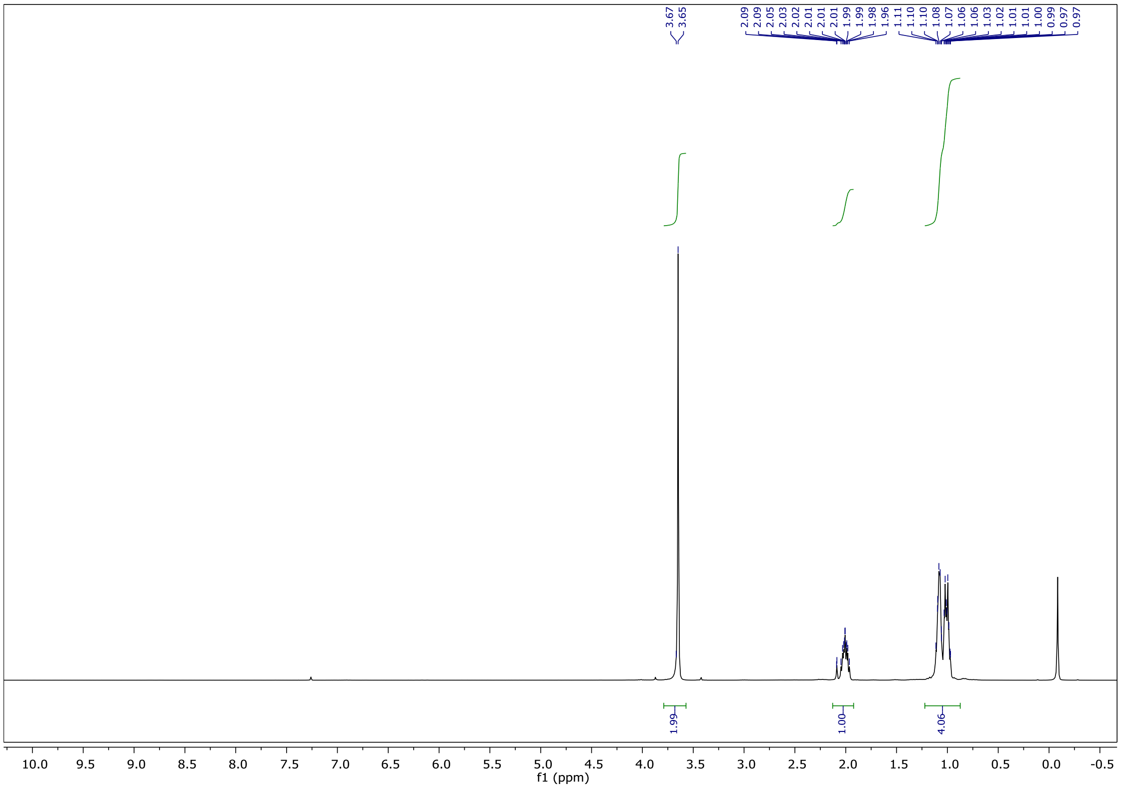

^{13}C NMR for 9 ($CDCl_3$, 75 MHz)

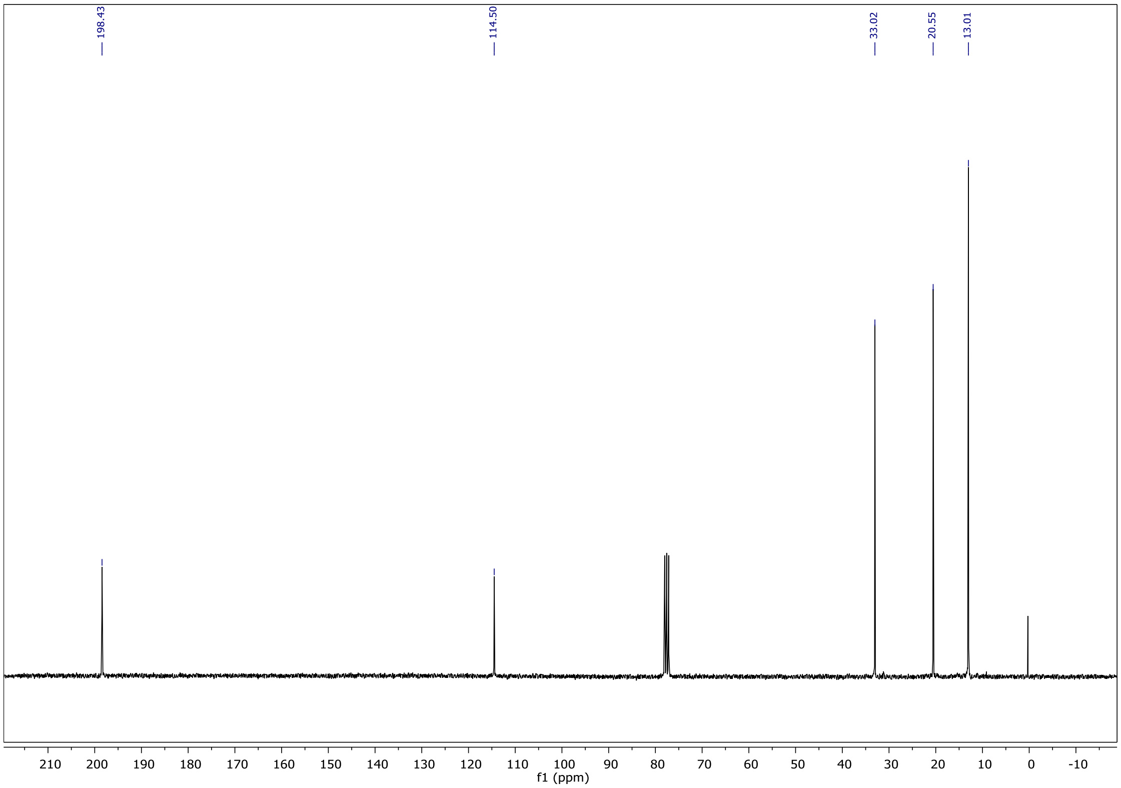

^{1}H NMR for 10 ($CDCl_3$, 300 MHz)

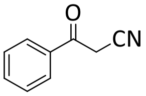

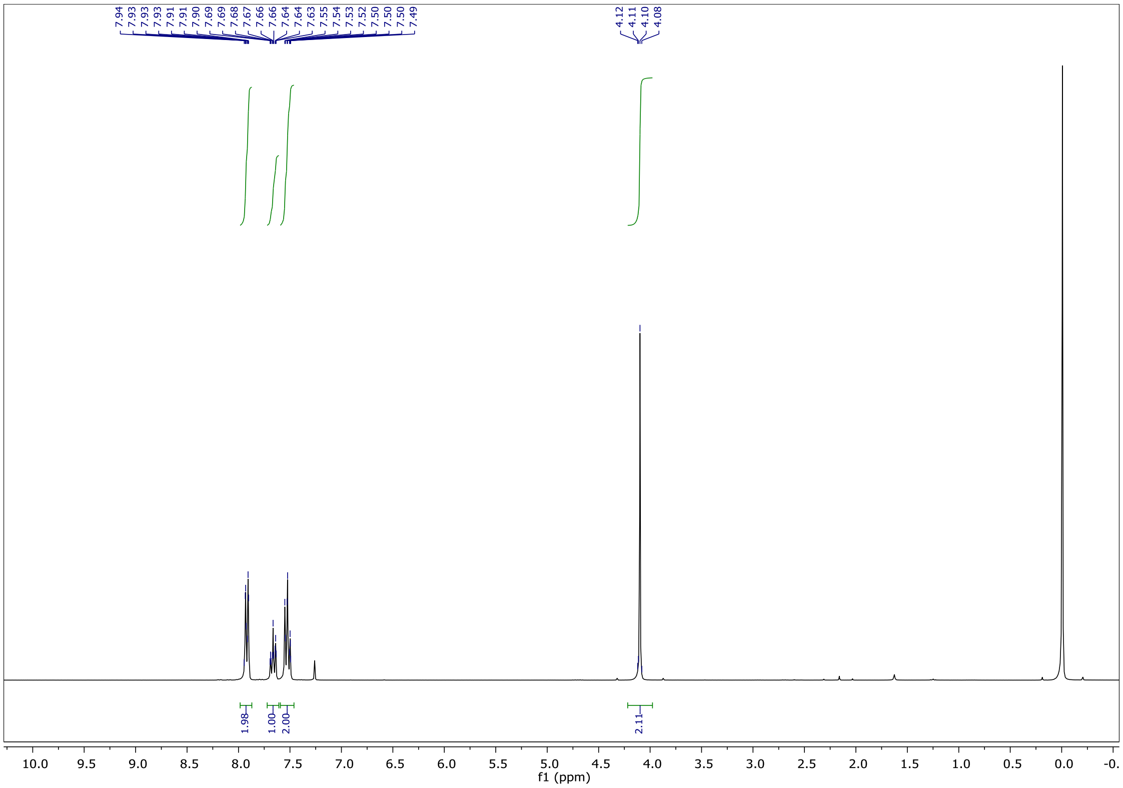

^{13}C NMR for 10 ($CDCl_3$, 75 MHz)

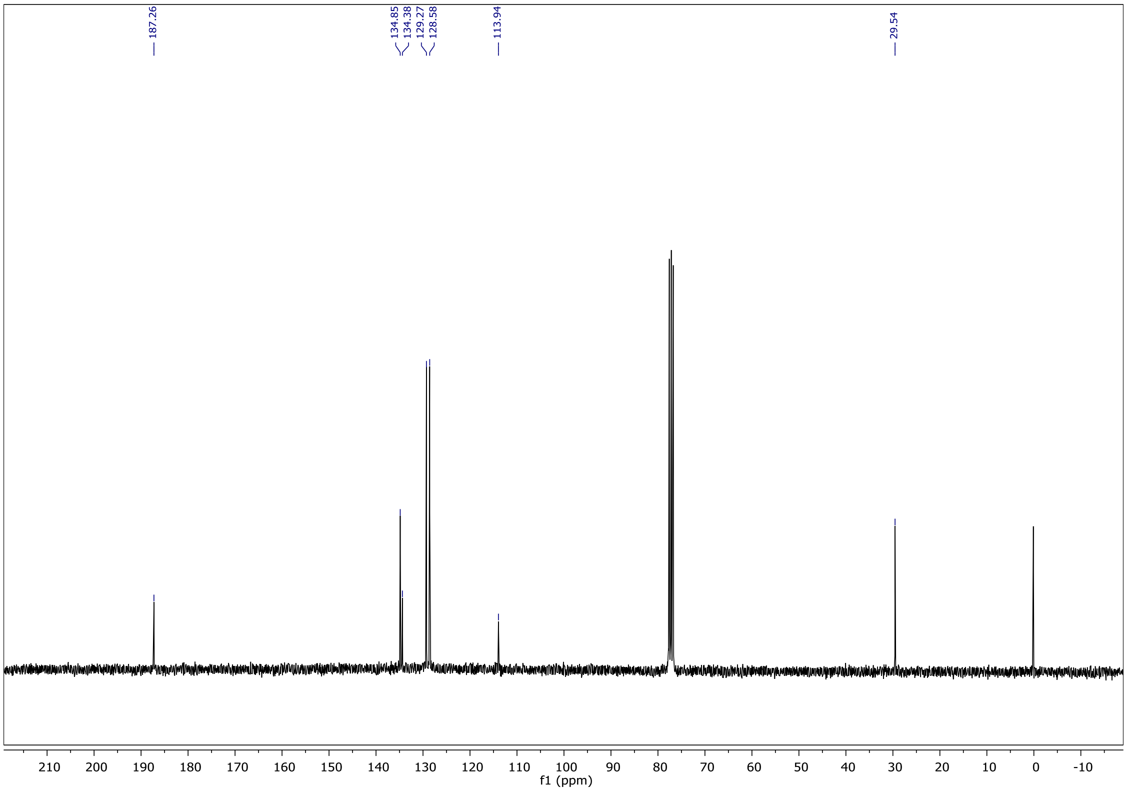

^{1}H NMR for 11 ($CDCl_3$, 400 MHz)

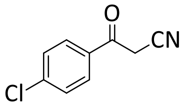

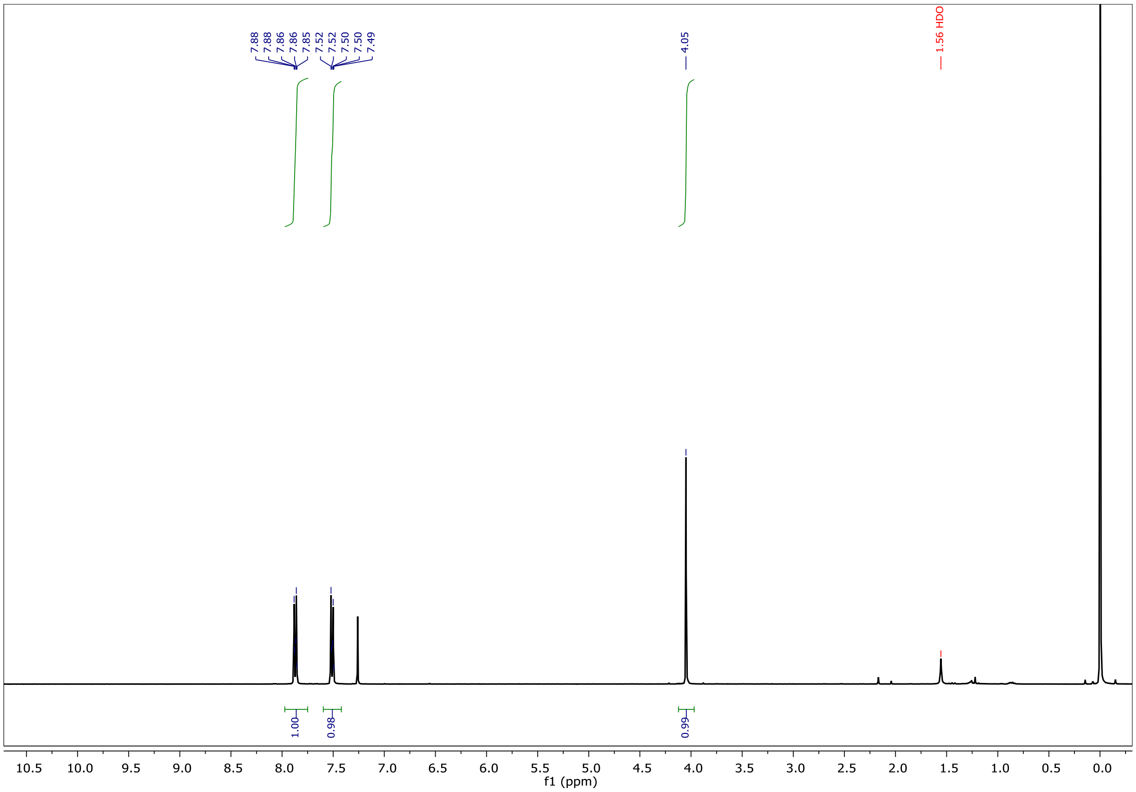

^{13}C NMR for 11 ($CDCl_3$, 101 MHz)

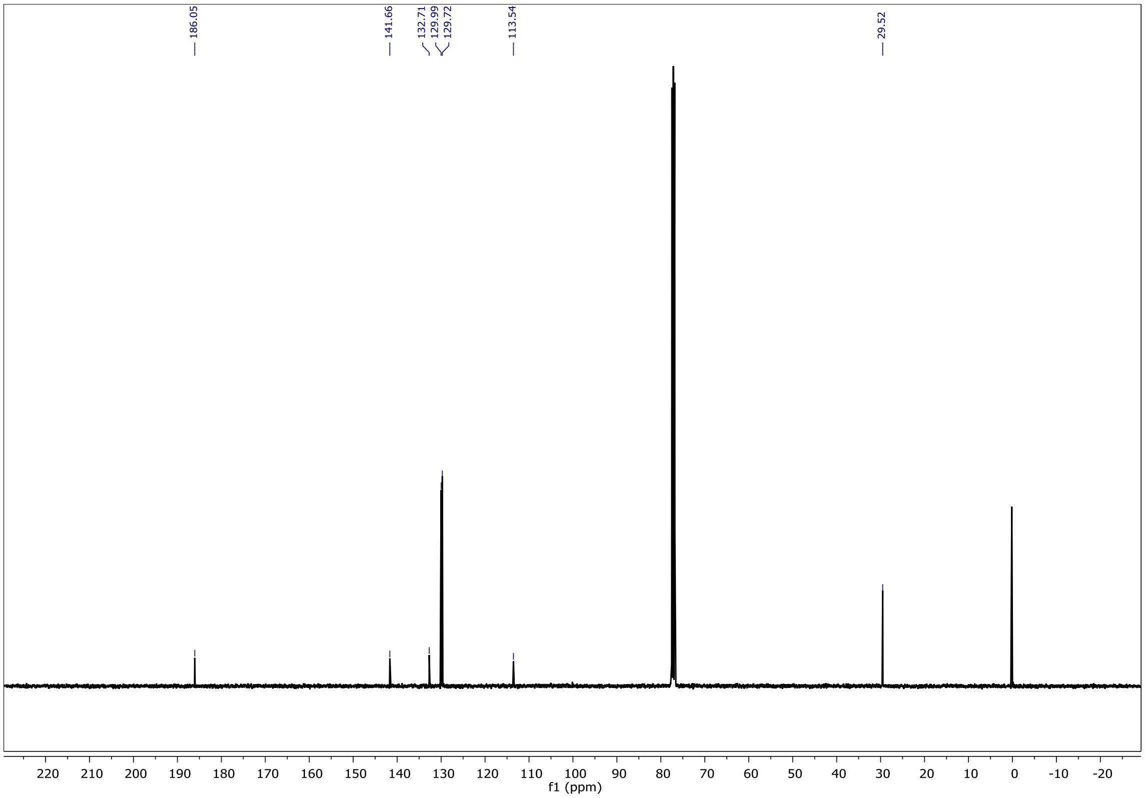

^{1}H NMR for 12 ($CDCl_3$, 300 MHz)

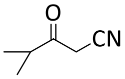

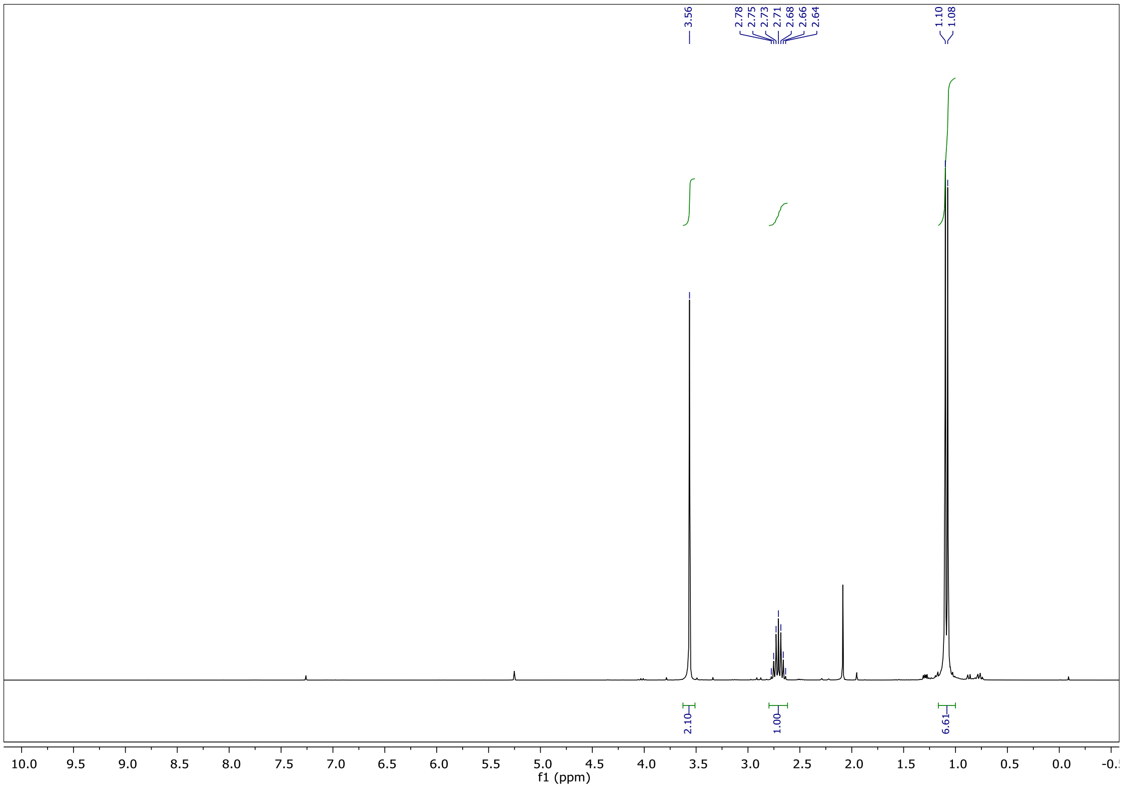

^{13}C NMR for 12 ($CDCl_3$, 75 MHz)

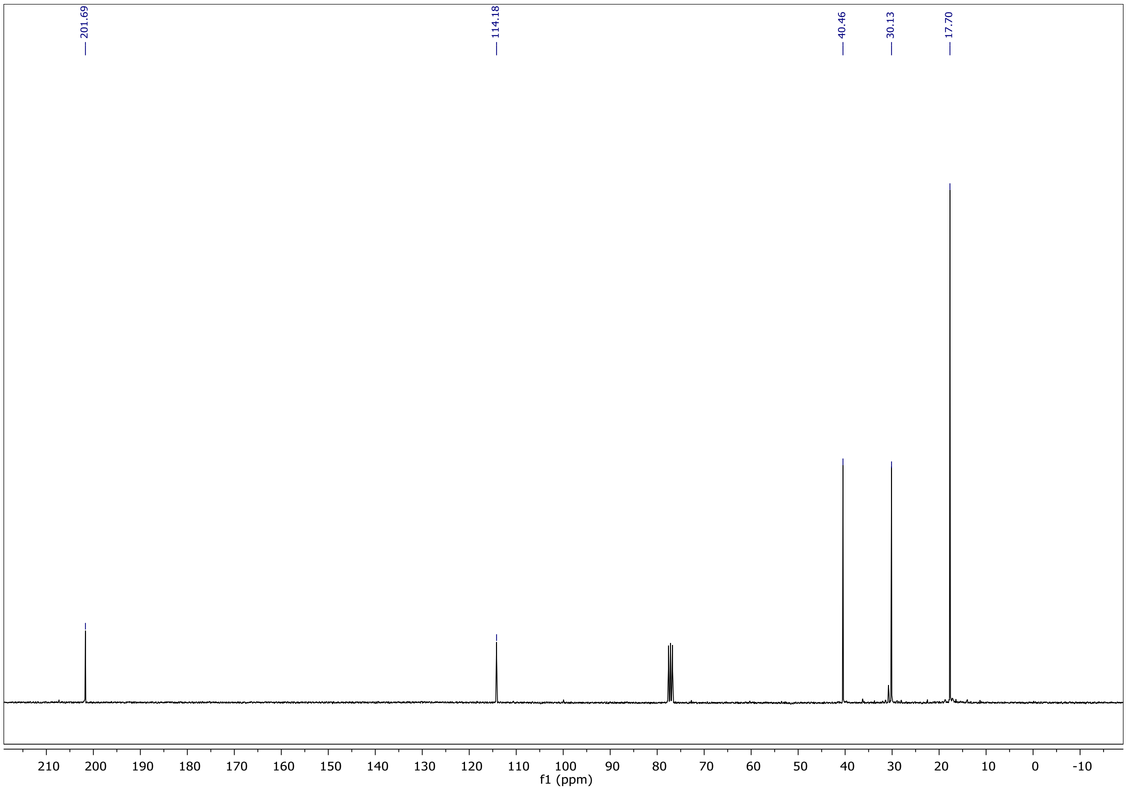

^{1}H NMR for 13 ($CDCl_3$, 300 MHz)

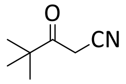

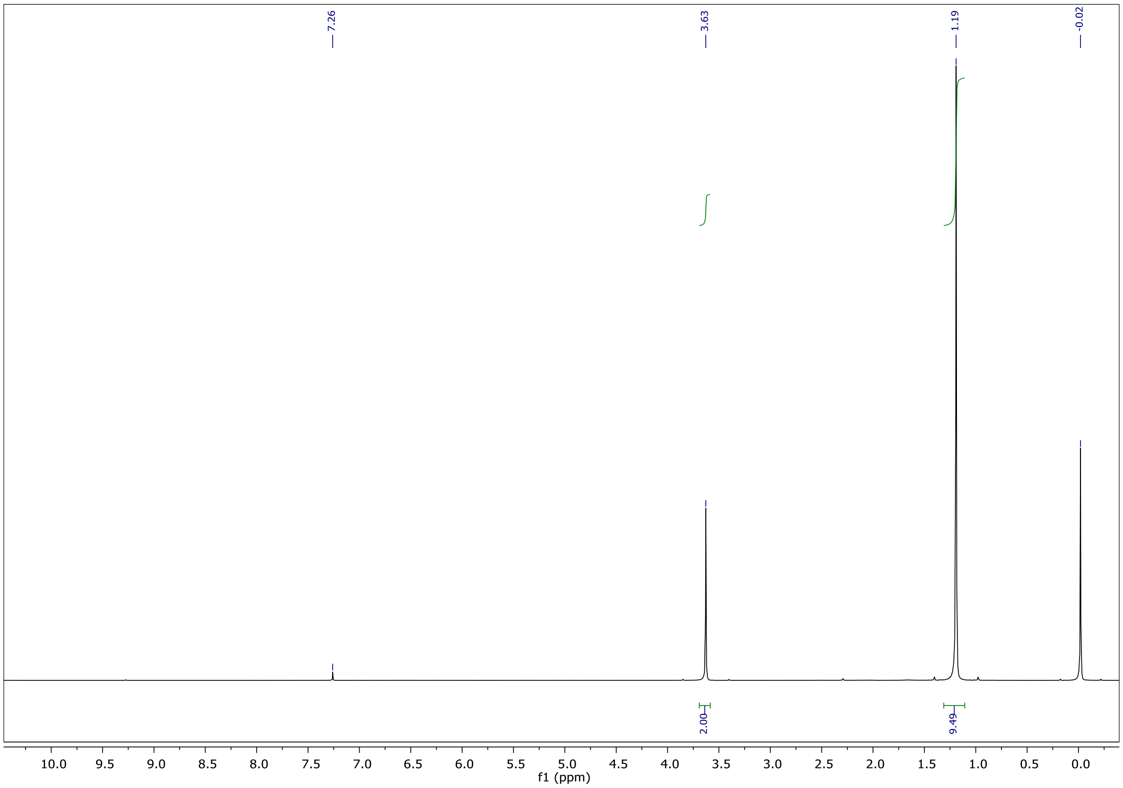

^{13}C NMR for 13 ($CDCl_3$, 75 MHz)

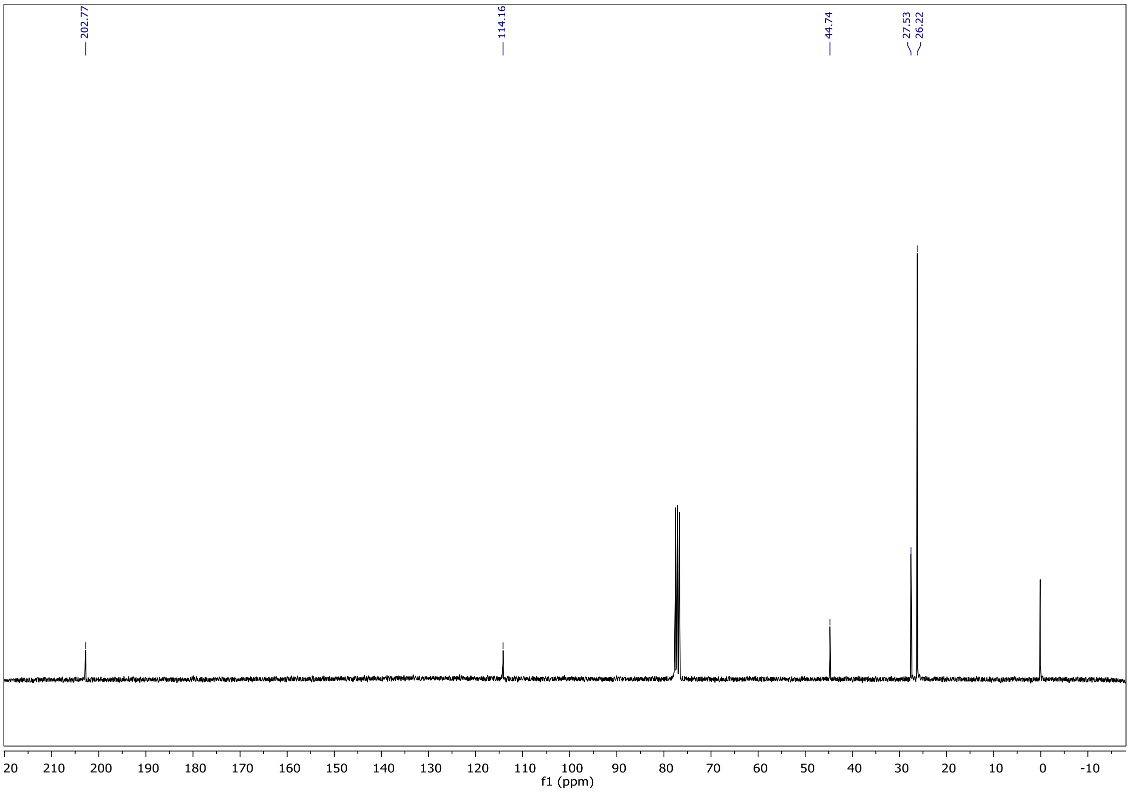

^{1}H NMR for 14 ($CDCl_3$, 300 MHz)

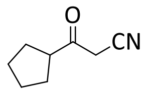

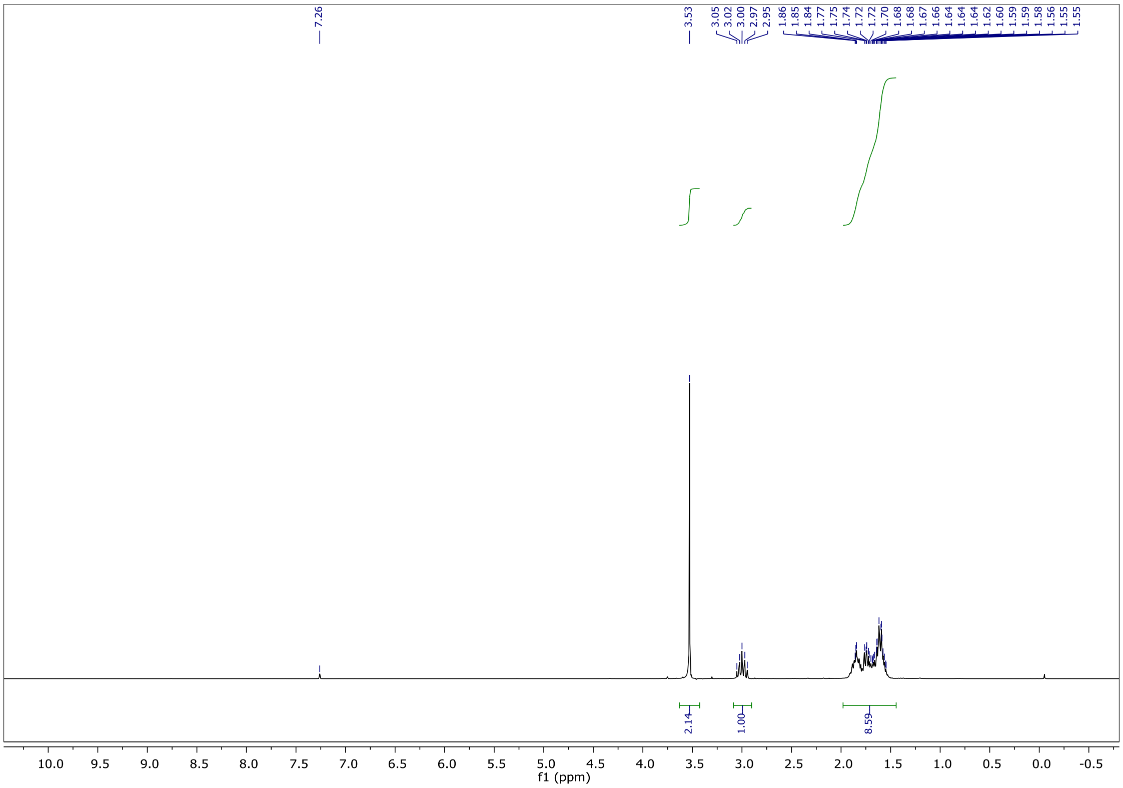

^{13}C NMR for 14 ($CDCl_3$, 75 MHz)

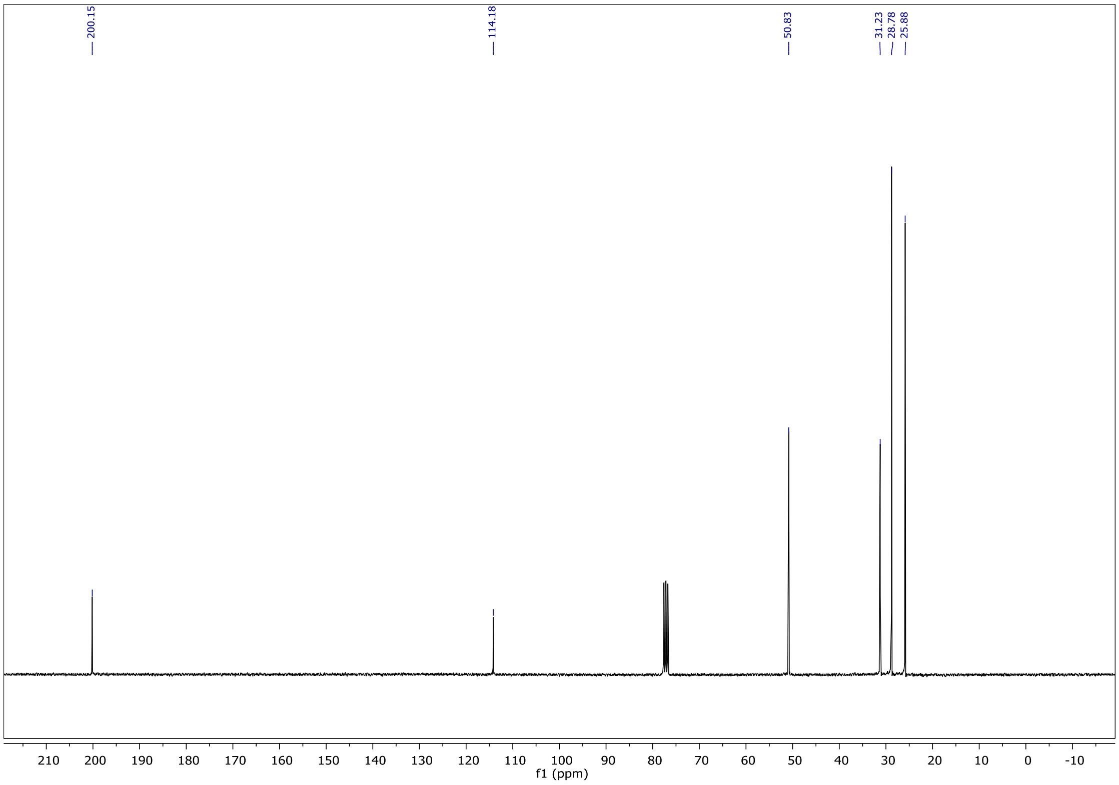

^{1}H NMR for 15 ($CDCl_3$, 300 MHz)

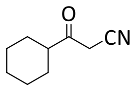

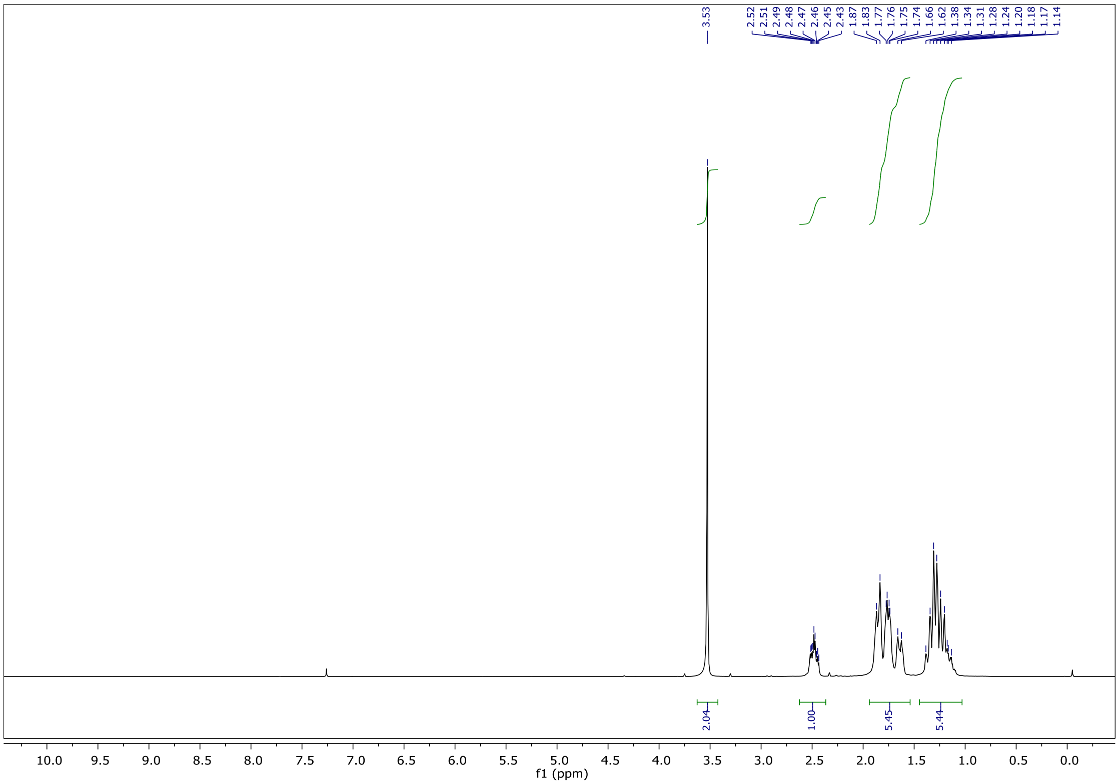

^{13}C NMR for 15 ($CDCl_3$, 75 MHz)

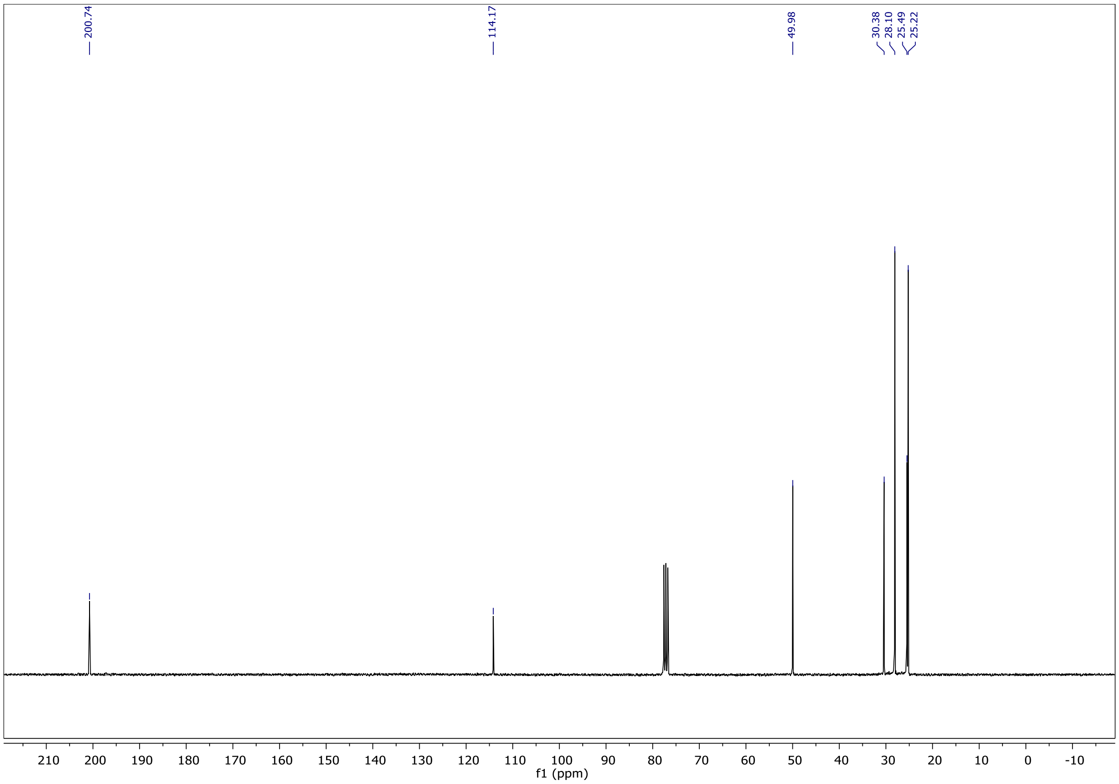

^{1}H NMR for 16 ($CDCl_3$, 300 MHz)

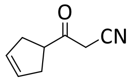

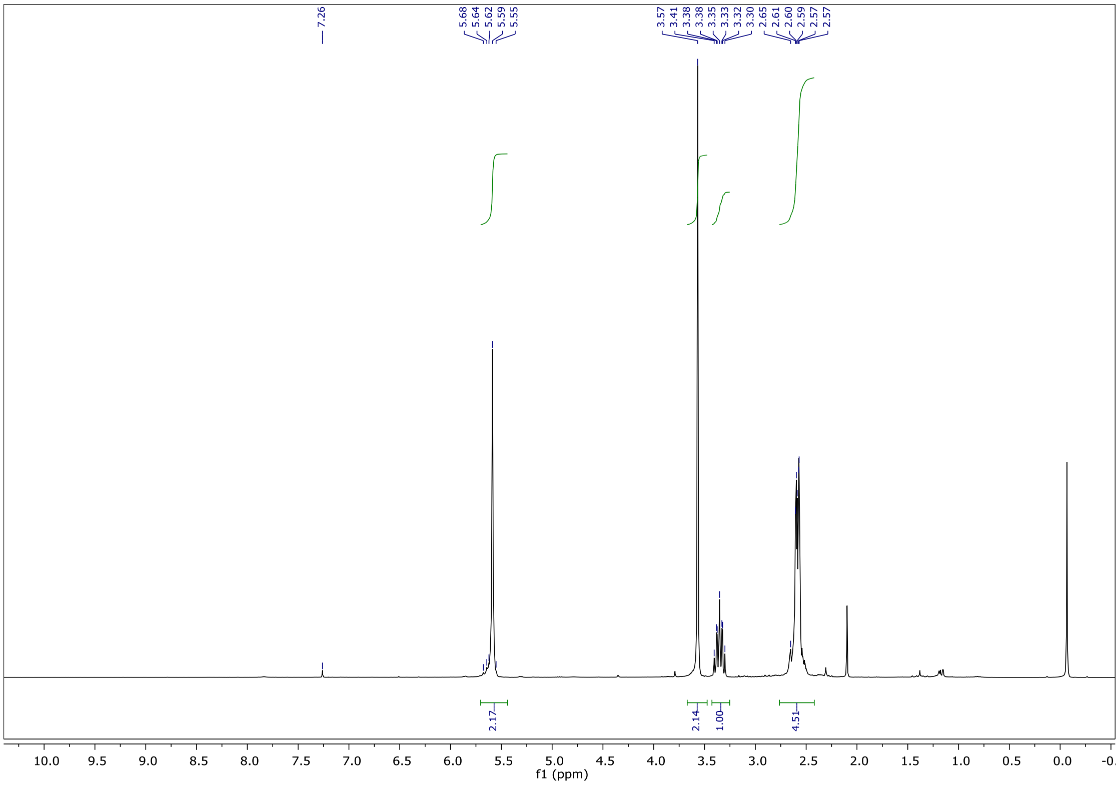

^{13}C NMR for 16 ($CDCl_3$, 75 MHz)

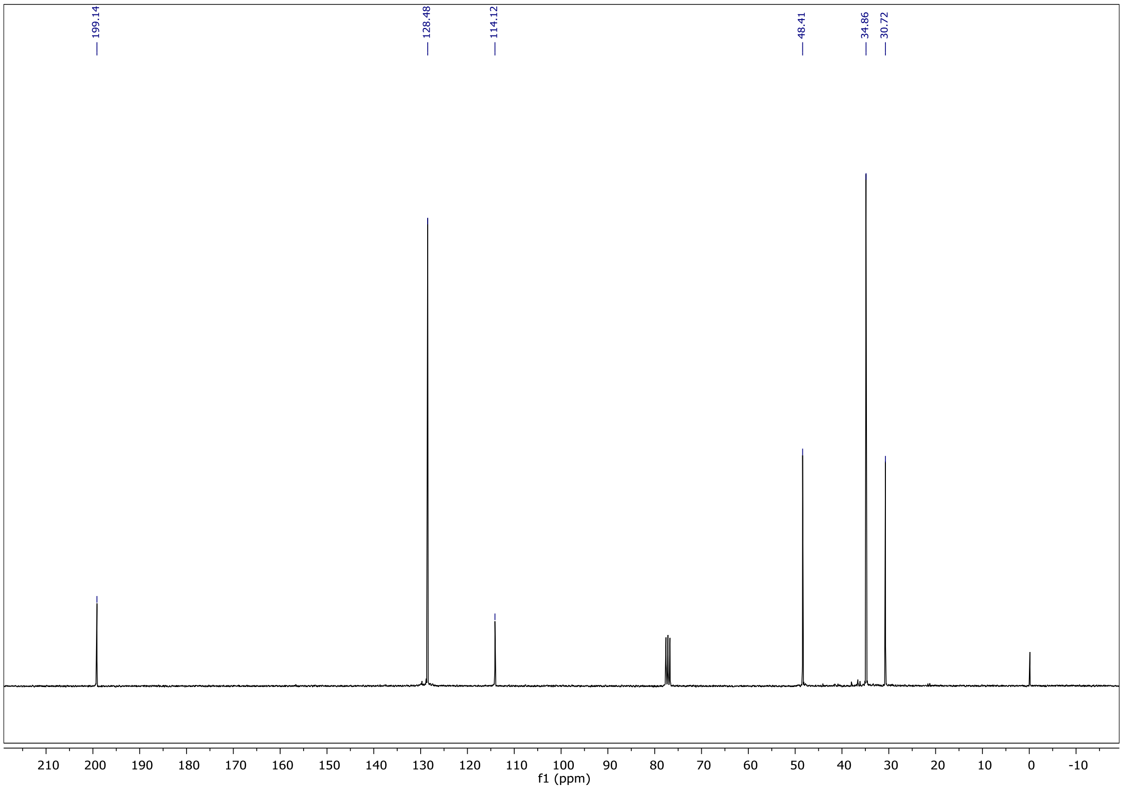

^{1}H NMR for 17 (CDCl$_3$, 300 MHz)

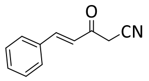

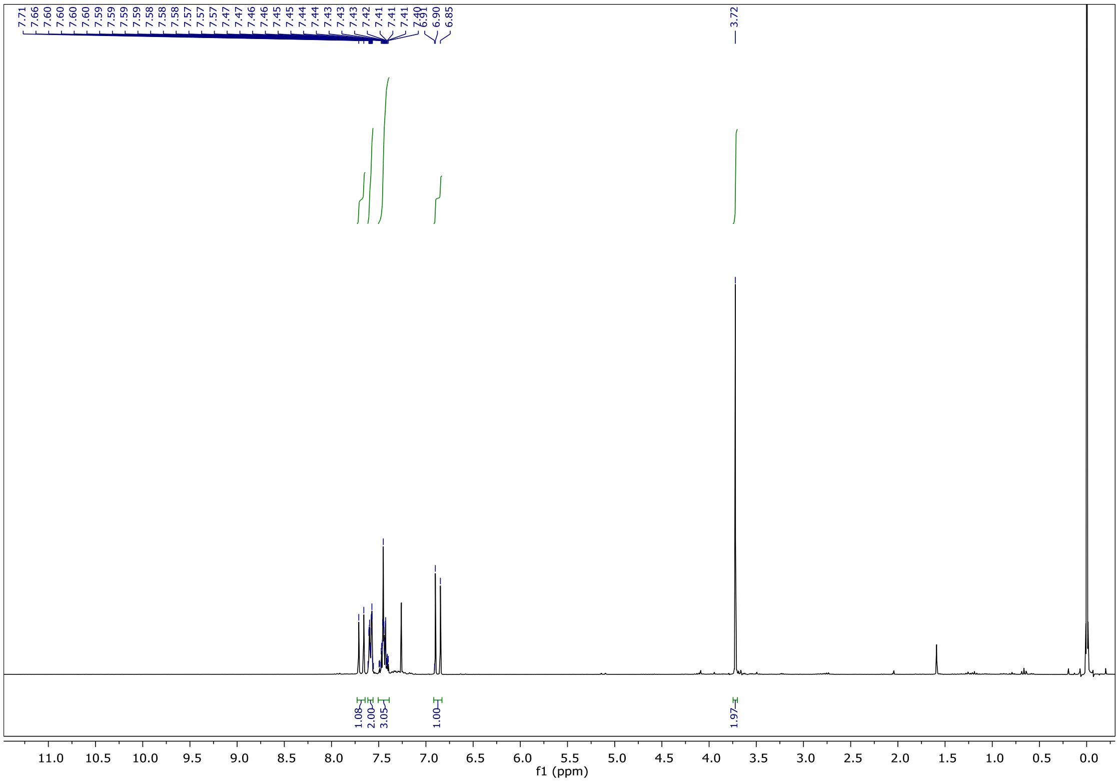

^{13}C NMR for 17 (CDCl$_3$, 75 MHz)

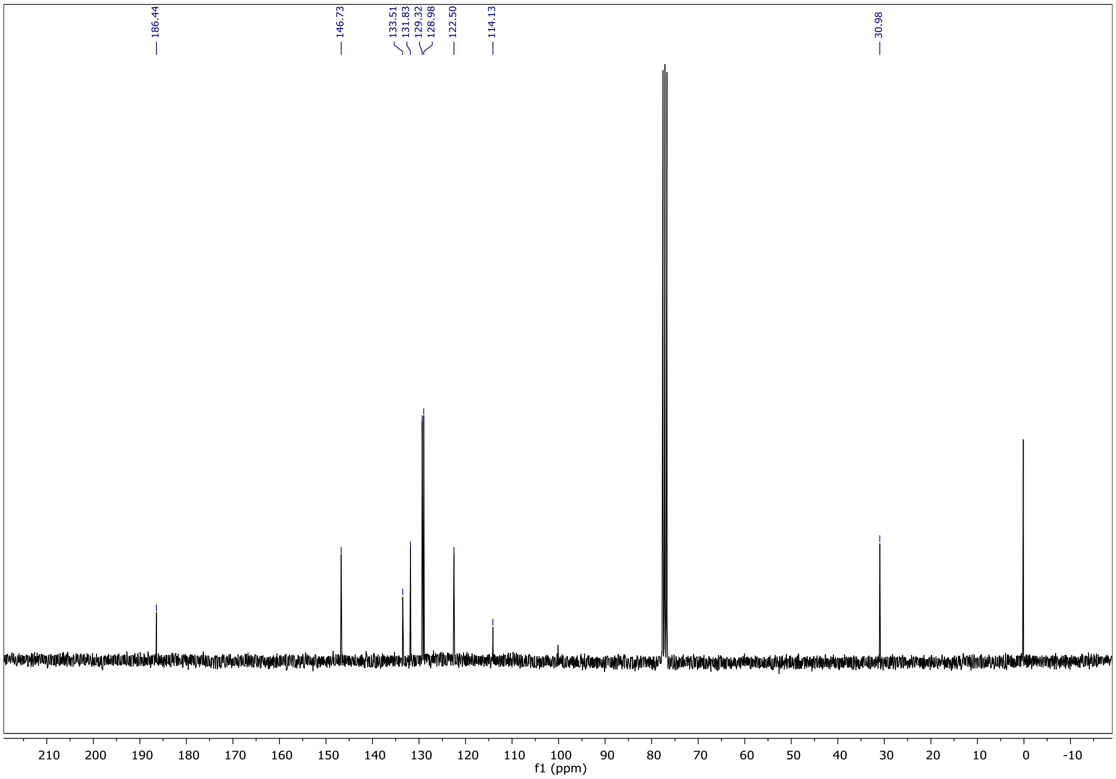

^{1}H NMR for methyl thiophene-2-carboxylate ($CDCl_3$, 300 MHz)

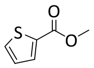

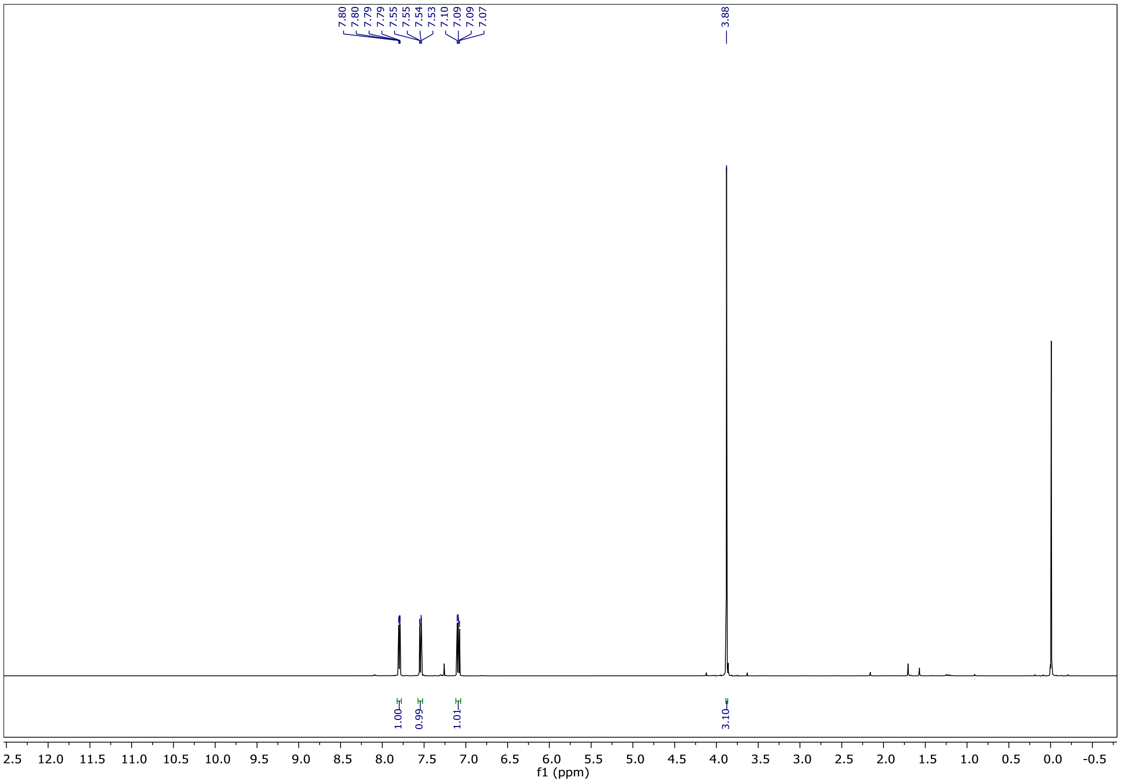

^{13}C NMR for methyl thiophene-2-carboxylate ($CDCl_3$, 75 MHz)

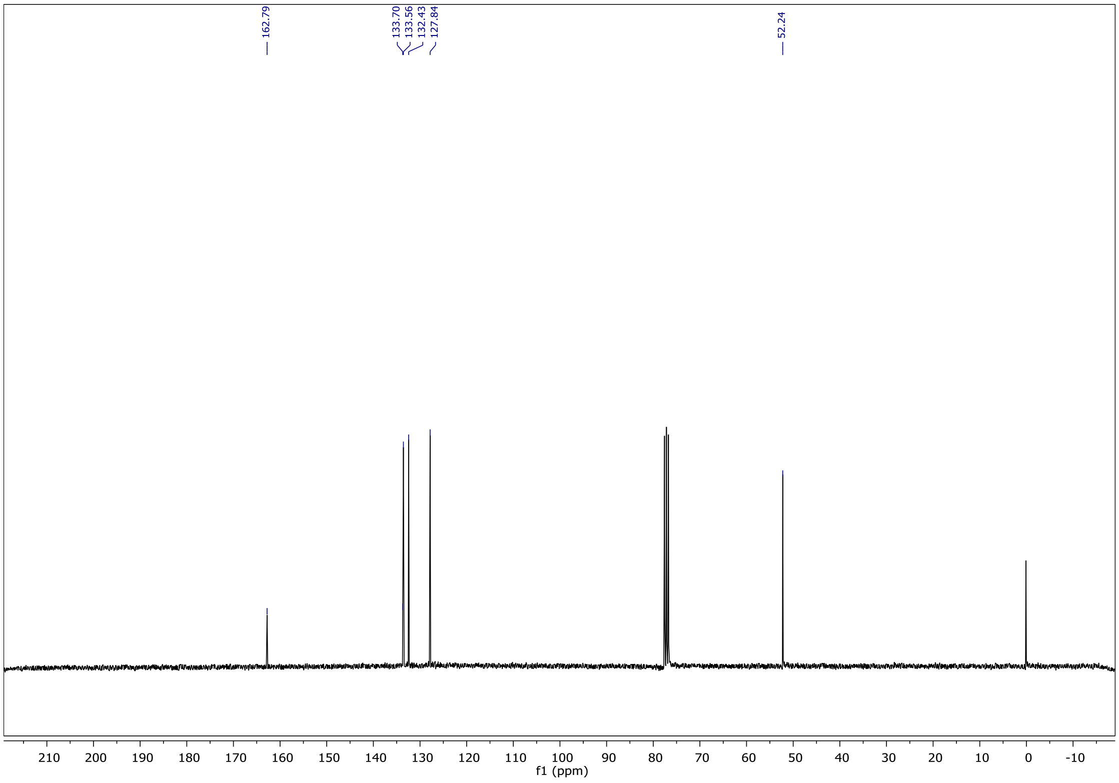

^{1}H NMR for 18 ($CDCl_3$, 300 MHz)

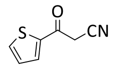

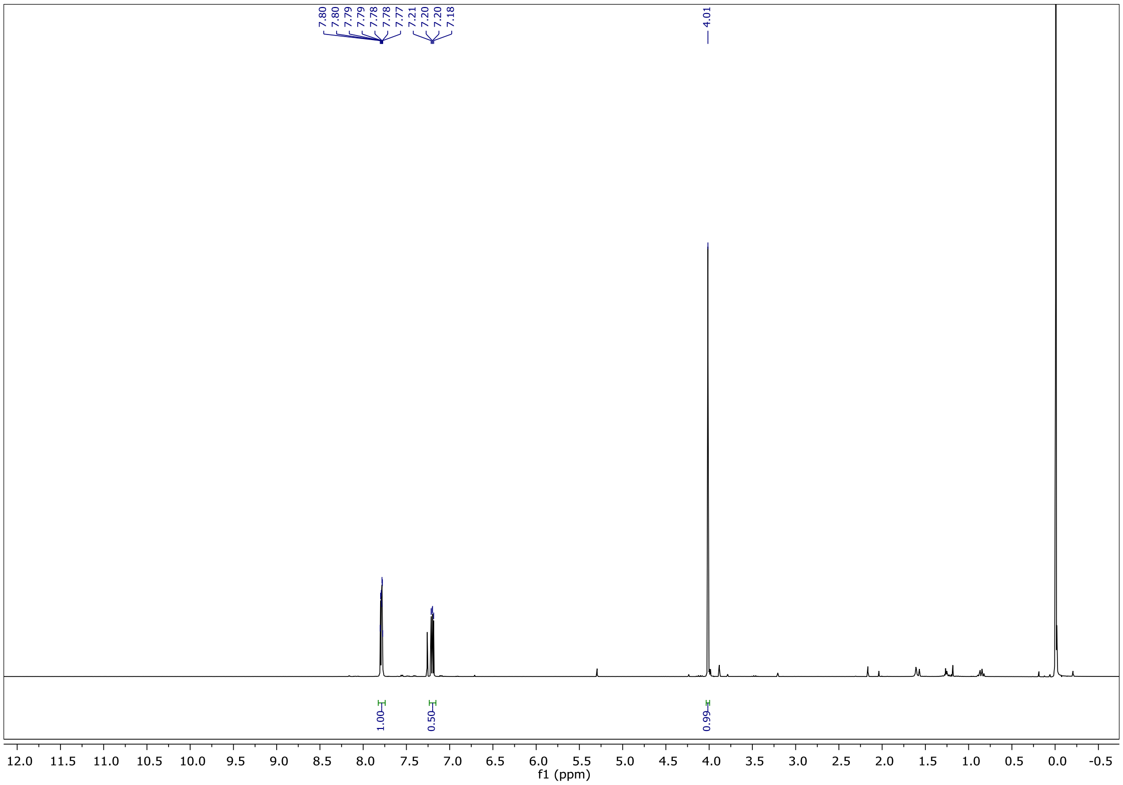

^{13}C NMR for 18 ($CDCl_3$, 75 MHz)

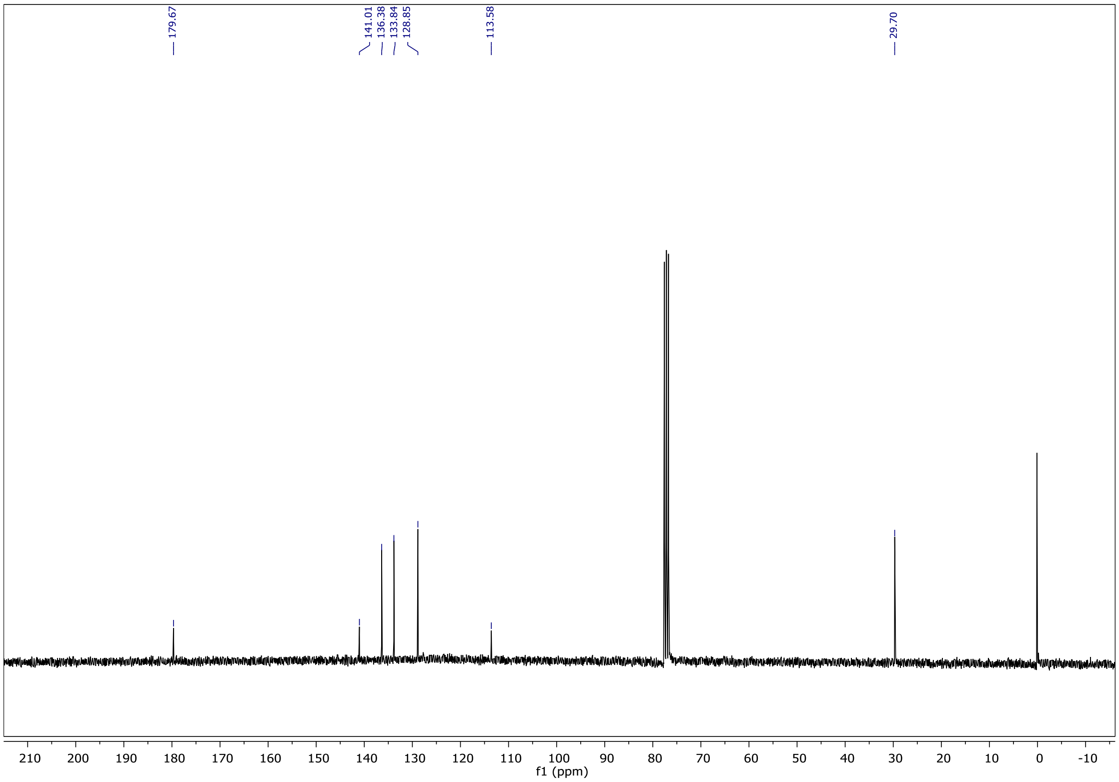

Supplement: File 1 — Experimental procedures, compound characterization data and NMR spectra. [file Beilstein_J_Org_Chem-15-2930-s001.pdf]
